# A Comprehensive Review of Surface Acoustic Wave-Enabled Acoustic Droplet Ejection Technology and Its Applications

**DOI:** 10.3390/mi14081543

**Published:** 2023-07-31

**Authors:** Jia Ning, Yulin Lei, Hong Hu, Chenhui Gai

**Affiliations:** School of Mechanical Engineering and Automation, Harbin Institute of Technology, Shenzhen 518055, China; 20b353003@stu.hit.edu.cn (J.N.);

**Keywords:** surface acoustic wave (SAW), acoustic droplet ejection (ADE), jetting

## Abstract

This review focuses on the development of surface acoustic wave-enabled acoustic drop ejection (SAW-ADE) technology, which utilizes surface acoustic waves to eject droplets from liquids without touching the sample. The technology offers advantages such as high throughput, high precision, non-contact, and integration with automated systems while saving samples and reagents. The article first provides an overview of the SAW-ADE technology, including its basic theory, simulation verification, and comparison with other types of acoustic drop ejection technology. The influencing factors of SAW-ADE technology are classified into four categories: fluid properties, device configuration, presence of channels or chambers, and driving signals. The influencing factors discussed in detail from various aspects, such as the volume, viscosity, and surface tension of the liquid; the type of substrate material, interdigital transducers, and the driving waveform; sessile droplets and fluid in channels/chambers; and the power, frequency, and modulation of the input signal. The ejection performance of droplets is influenced by various factors, and their optimization can be achieved by taking into account all of the above factors and designing appropriate configurations. Additionally, the article briefly introduces the application scenarios of SAW-ADE technology in bioprinters and chemical analyses and provides prospects for future development. The article contributes to the field of microfluidics and lab-on-a-chip technology and may help researchers to design and optimize SAW-ADE systems for specific applications.

## 1. Introduction

Microscale droplets, with diameter dimensions spanning from nanometers to micrometers, have significant utilization in diverse fields including bioprinting, chemical synthesis, industrial dispensing, and beyond. To generate and deliver microdroplets to target locations, different approaches have been developed on a basis of different physical principles. Some of common methods are inkjet, pneumatic jetting [1], piezoelectric jetting [2], electro-hydrodynamic jetting [3], and laser induction jetting [4]. However, these methods have some limitations, such as the clogging of nozzles, damage of biological materials because of high temperature or electric fields, low throughput or resolution due to limited frequency or size of droplets, etc. One of the most promising methods is acoustic droplet ejection (ADE), which utilizes acoustic waves to manipulate liquids without physical contact or external heating.

As a non-contact method, ADE aims to transfer small amounts of fluid. The ADE-based liquid handling method eliminates cross-contamination and is not limited by the nozzle design. This technology has brought improvements to various fields, such as drug discovery, genomics, proteomics, and personalized medicine [5]. Although ADE technology has been widely studied as a fluid transferring method, it has limitations in terms of droplet volume, frequency, and stability. However, with the development of acoustic driving devices and automated power control solutions, the ADE system can now dispense droplets stably and quickly. 

A surface acoustic wave (SAW)-enabled acoustic droplet ejection is one type of acoustic jetting that can generate a single droplet with high precision and repeatability by focusing acoustic energy on liquid using specially designed devices. SAW is a type of elastic wave that propagates along the surface of an elastic medium with most of its energy confined within one wavelength depth below the surface. The existence of SAW was first discussed by Lord Rayleigh in 1885 who derived its dispersion relation for isotropic elastic media [6] but did not find any practical applications until 1965 when White and Voltmer demonstrated the generation and detection of SAW using interdigital transducers (IDTs) deposited on piezoelectric substrates, such as quartz or lithium niobate [7]. SAW can be classified into different modes according to its polarization direction, such as Rayleigh SAW (R-SAW) [8], leaky SAW (L-SAW) [9], shear-horizontal SAW (SH-SAW) [10] and lamb wave [11]. In the layered structures, Love waves, Sezawa waves, Stoneley waves, and other waveforms may exist in the substrate [12]. Due to its low velocity (about 3000 m/s for quartz or 3990 m/s for LiNbO_3_) compared to electromagnetic waves and high frequency sensitivity [13] (up to GHz range), SAW devices were initially widely used as filters in telecommunication systems [14]. Later, it was also applied as sensors for various physical quantities, such as temperature, pressure, strain, mass, etc. [15]. With the development of microfluidics technology, SAW devices were also applied as actuators for controlling microfluids microdroplets and particles in fluids using various ways depending on the input power and frequency of electrical signals, the geometry and material of SAW devices and substrates, the properties and configuration of liquids and particles, etc.

SAW devices can exert pressure forces on liquid due to acoustic radiation pressure (ARP), resulting from momentum transfer between acoustic waves and liquids. They can also induce capillary waves on liquid due to acoustic streaming (AS) resulting from viscous drag. SAW devices are able to generate strong acoustic fields on liquid due to high acoustic impedance mismatch between solid and liquid media. In the past, the cavitation in high-frequency SAW was negligible due to the onset of cavitation was determined by the square of the frequency [16,17,18,19]. Recent evidence indicates the occurrence of microcavitation in SAW aerosol generation platforms operating within the MHz frequency range. Moreover, it has been observed that this microcavitation phenomenon leads to surface erosion during SAW operations [20]. 

SAWs are frequently employed as acoustic tweezers [21] in particles and cell manipulation. Utilizing ARP, AS, or both, SAWs can be used for a variety of manipulation techniques, including particles and cell manipulation [22,23], trapping [24], sorting [25], patterning [26,27], concentration [28,29], separation [30,31], coating [32], etc. These techniques have demonstrated significant potential for SAW-based applications in fields, such as biosensing, bioanalysis, and microfluidics.

In addition to their frequent use in particles and cell manipulation, SAWs are also commonly utilized for driving the trapping [33] and splitting [34] of liquid droplets in channels as well as for the manipulation [35] of sessile droplets, which includes techniques such as translation [36], combination [37], pumping [38,39], mixing [40,41], merging [42], atomization [43,44,45,46], and jetting [12,47]. 

SAW technology offers a means for the generation of precise and reproducible single or multiple droplets through jetting. This is achieved by focusing acoustic energy on liquid surfaces using specialized ITDs, resulting in a method called SAW-ADE. SAW-ADE overcomes liquid surface tension by creating a pressure gradient that exceeds the Rayleigh-Taylor instability criterion. The gradient induces a liquid protrusion that transforms into a jet and ultimately breaks into single or multiple droplets due to Plateau–Rayleigh instability. SAW-ADE presents several advantages over other microdroplet generation and delivery methods, including low temperature and electric field for excellent biocompatibility, remote excitation for non-contact operation, nozzle-free design resulting from surface wave propagation, planar structure facilitating easy integration with microfluidic technology, and high throughput and resolution owing to the high frequency range.

SAW-ADE technology exhibits potential for producing microdroplets in a precise and contactless manner by utilizing SAWs. It has gained increasing attention recently due to its substantial application in a wide range of fields, including biomedicine, chemistry, and materials science. Despite considerable progress in experimental studies, the underlying mechanism of droplet ejection under the joint action of SAWs and microfluidics remains inadequately understood. Consequently, a methodical theoretical framework for comprehending the ejection mechanism has yet to be established. Moreover, the absence of a standard protocol for employing different simulation tools has hindered research results’ comparability and reproducibility. Additionally, the practical reliability of SAW-ADE technology remains insufficiently addressed. In particular, the influence of various environmental factors, such as temperature, humidity, and atmospheric pressure, on the droplet ejection process also necessitates further investigation. Addressing these challenges is crucial for advancing SAW-ADE technology and its successful integration into practical applications. This review provides a comprehensive summary of the fundamental theory, influencing factors, and potential applications of SAW-ADE technology. The article first introduces the basic principles and simulation validation of SAW-ADE, including the acoustic streaming theory, SAW-induced jetting, and simulation verification. The formation of droplet jetting is affected by various factors, such as fluid properties, device configuration, presence of channels or chambers, and driving signals. To generate SAW jetting, the mother droplet size must be larger than the SAW wavelength. The article further discusses different types of IDTs and substrate materials used in SAW-ADE as well as the effect of substrate treatment with hydrophobic materials to improve driving capability. Moreover, the different types of SAWs that can be generated and utilized for microfluidic manipulation are explored. The article concludes by discussing various ejection scenarios that can occur depending on the presence of channels or chambers. The parameter configuration of driving signals is also summarized. Finally, the review highlights the potential applications of SAW-enabled ADE in bioprinters and chemical analysis.

## 2. Fundamentals of ADE

### 2.1. Acoustic Streaming Theory 

When SAWs propagate on the substrate surface and come into contact with a droplet, the SAWs attenuate to leaky surface acoustic waves (LSAWs) due to the mismatch in velocity. The LSAWs continue to attenuate in the liquid, and their attenuation coefficient $\alpha_{l}\;$ can be expressed by the following equation [48,49]:

(1) $$\alpha_{l}=\frac{1}{l_{\mathrm{SAW}}}=\frac{\rho u}{\rho_{s}u_{s}\lambda }=\frac{\rho uf}{\rho_{s}u_{s}^{2}}$$

where $\rho_{s}$ and ρ represent the densities of the LiNbO_3_ substrate and the fluid, respectively; u is the velocity of longitudinal wave in the fluid; $u_{s}$ is the SAW velocity in the substrate; λ is the SAW wavelength; f is the SAW frequency; and $l_{\mathrm{SAW}}$ is the attenuation length of the Rayleigh wave.

Simultaneously, the diffracted longitudinal wave enters the droplet at the Rayleigh angle $\theta_{R}=\arcsin\left(u/u_{s}\right)$, producing a pressure gradient and causing fluid flow, further generating an acoustic streaming. The acoustic radiation pressure ($P_{s}$) builds up in the direction of the longitudinal wave propagation in the fluid can be expressed as [50]:

(2) $$P_{s}=\rho u_{s}^{2}{\left(\frac{\Delta \rho }{\rho }\right)}^{2}$$

Here, Δρ represents the density change caused by the sound pressure.

As the amplitude rises, the droplet undergoes a variety of dynamic changes, such as jetting, vibration, driving, and atomization, which are collectively referred to as SAW acoustic streaming. The theory of SAW acoustic streaming is derived from Nyborg’s acoustic streaming theory [51], which is mainly guided by the incompressible Navier–Stokes equation and the continuity equation. Acoustic streaming is mainly driven by volume forces and for general hydrodynamic viscous fluids:

(3) $$F=\rho \frac{Du}{Dt}=\rho \frac{\partial u}{\partial t}+\rho \left(u\cdot \nabla \right)u=-\nabla p+\mu \nabla^{2}u+\left(\mu_{B}+\frac{\mu }{3}\right)\nabla \left(\nabla \cdot u\right)$$

where F is the net force per unit volume, p is the fluid pressure, μ is the shear viscosity of the fluid, and $\mu_{B}$ is the bulk dynamic viscosity of the fluid. The continuity equation for fluid can be expressed as follows:

(4) $$\frac{\partial \rho }{\partial t}+\nabla \cdot \left(\rho u\right)=0$$

Combining Equations (3) and (4):


(5) $$\frac{\partial \rho u}{\partial t}+\rho \left(u\cdot \nabla \right)u+u\nabla \cdot \rho u=-\nabla p+\mu \nabla^{2}u+\left(\mu_{B}+\frac{\mu }{3}\right)\nabla \left(\nabla \cdot u\right)$$

Definition:


(6) $$F_{0}=\frac{\partial \rho u}{\partial t}$$

(7) $$-F_{1}=\rho \left(u\cdot \nabla \right)u-u\nabla \cdot \rho u$$

By utilizing perturbation methods, the fluid field of pressure, density, and velocity can be expanded as:

(8a) $$u=\varepsilon u_{1}+\varepsilon^{2}u_{2}+O\left(\varepsilon^{3}\right)+\cdots$$

(8b) $$p=p_{0}+\varepsilon p_{1}+\varepsilon^{2}p_{2}+O\left(p^{3}\right)+\cdots$$

(8c) $$\rho =\rho_{0}+\varepsilon \rho_{1}+\varepsilon^{2}\rho_{2}+O\left(\rho^{3}\right)+\cdots$$

The subscript 0 denotes the quantity in the static state, subscript 1 represents the first-order quantity of the acoustic field, and subscript 2 represents the second-order quantity of the acoustic field. The expression ε≪1 is defined as a non-dimensional small parameter characterizing the order of the acoustic response.

Substituting Equation (8) into Equations (4) and (5) yields first-order and second-order field equations. The continuity equation and motion equation for the first-order field are expressed as:

(9) $$\frac{\partial \rho_{1}}{\partial t}+\rho_{0}\left(\nabla \cdot u_{1}\right)=0$$

(10) $$\rho_{0}\frac{\partial u_{1}}{\partial t}=-\nabla \rho_{1}+\mu \nabla^{2}u_{1}+\left(\mu_{B}+\frac{\mu }{3}\right)\nabla \left(\nabla \cdot u_{1}\right)$$

The aforementioned first-order equations determine the acoustic motion of the system. The acoustic solution $u_{1}$ can be used to determine the second-order acoustic flow motion. The continuity equation and motion equation for the second-order acoustic flow fields are as follows:

(11) $$\frac{\partial \rho_{2}}{\partial t}+\nabla \cdot \left(\rho_{0}u_{2}\right)+\nabla \cdot \left(\rho_{1}u_{1}\right)=0$$

(12) $$\rho_{0}\frac{\partial u_{2}}{\partial t}+\rho_{0}\left(u_{1}\cdot \nabla \right)u_{1}+\rho_{1}\frac{\partial u_{1}}{\partial t}=-\nabla p_{2}+\mu \nabla^{2}u_{2}+\left(\mu_{B}+\frac{\mu }{3}\right)\nabla \nabla \cdot u_{2}$$

To obtain the acoustic flow equation, we retain these second-order terms and take their averages over a suitable number of cycles. In a steady state, the time average of $F_{0}$ is zero, and Equation (7) can be expressed as:

(13) $$-F_{1}=\langle \rho_{0}\left(u\cdot \nabla \right)u-u\nabla \cdot \rho_{0}u\rangle$$

in which the symbol 〈X〉 means “time average of *X*”. Considering the propagation of an attenuated plane wave in a fluid without boundaries, the displacement of fluid particles ($d_{x},d_{z})\;$can be expressed by the following equation [9,52]:

(14a) $$d_{x}=A\exp\left(j\omega t\right)\cdot \exp\left(-jk_{L}x\right)\cdot \exp\left(-\alpha k_{L}z\right)$$

(14b) $$\;d_{z}=-j\alpha A\exp\left(j\omega t\right)\cdot \exp\left(-jk_{L}x\right)\cdot \exp\left(-\alpha k_{L}z\right)$$

Here, $\alpha^{2}={\left(1-u_{s}/u\right)}^{2}$, *A* represents the amplitude of the SAW while ω is angular frequency of sound wave. The complicated wave number of the LSAWs, $k_{L}$ can be calculated by extending the method of Campbell and Jones [53] to liquid/solid structures, assuming that the boundary conditions for both displacement and stress at *z* = 0 are continuous. The effect of viscosity is neglected in Equation (14). By replacing the particle displacement in Equation (14) with particle velocity, i.e., u=dd/dt, substituting the particle velocity into Equation (13). $F_{x}$ and $F_{z}$ are given by:

(15a) $$F_{x}=-\rho_{0}\left(1+\alpha_{1}^{2}\right)A^{2}\omega^{2}k_{i}\exp2\left(k_{i}x+\alpha_{1}k_{i}z\right)$$

(15b) $$F_{z}=-\rho_{0}\left(1+\alpha_{1}^{2}\right)A^{2}\omega^{2}\alpha_{1}k_{i}\exp2\left(k_{i}x+\alpha_{1}k_{i}z\right)$$

Here, $\alpha =j\alpha_{1},\;k_{L}=k_{r}+jk_{i}$. The quantities $F_{x}$ and $F_{z}$ denote the components of the volume force in the *x* and *z* directions, respectively. Moreover, the streaming force $F_{ls}$ is expressed as a function of:

(16) $$F_{ls}=\sqrt{\left(F_{x}{}^{2}+F_{z}{}^{2}\right)}$$

So,

(17) $$F_{1s}=-\rho_{0}{\left(1+\alpha_{1}^{2}\right)}^{\frac{3}{2}}A^{2}\omega^{2}k_{i}\cdot \exp2\left(k_{i}x+\alpha_{1}k_{i}z\right)$$

The direction of $F_{1s}$ and the radiation of the LSAWs share the same angle. Furthermore, it has been observed that the amplitude of surface acoustic waves is directly proportional to the input radio frequency (RF) power and can be characterized in terms of the input RF power and wavelength using the following equation [48,54]:

(18) $$\frac{A}{\lambda }=8.15\times 10^{-6}P_{D}^{0.225}+5\times 10^{-6}P_{D}^{0.8}$$

where $P_{D}$ is the RF power applied to the SAW device in Watts.

### 2.2. Jetting Induced by SAW

Influenced by the surface acoustic wave streaming, the sessile droplet can be characterized by its streaming Reynolds number [47]:

(19) Res≡ρUsRdμ

where ρ and μ represent the liquid density and viscosity, respectively, and $U_{s}$ is the characteristic streaming velocity.

**Figure 1:**
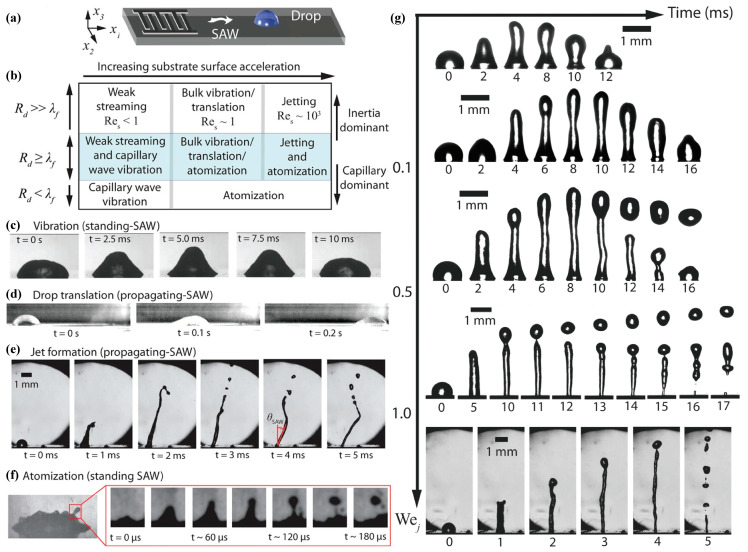
(**a**) Illustration of the SAW device on which a deionized water drop is placed in the propagation pathway of the SAW. (**b**) Summary of the different drop behaviors observed as a function of the LSAW wavelength and the drop size: (**c**) drop vibration, (**d**) translation, (**e**) jetting at the Rayleigh angle, and (**f**) atomization due to SAW. (**g**) The transition of droplet jetting by increased the jet Weber number ${\mathrm{We}}_{j}$. Reprinted from Tan et al. [47].

At Res~1, $U_{s}\sim 0.001\;m/s$, the bulk vibration of the drop is observed under the influence of standing waves (Figure 1c), or the drop translation (Figure 1d) is observed due to propagating surface acoustic waves in the droplet generates sufficient body force to overcome contact line pinning [55]. As the input power increases further, the inertial streaming (Res~1000) cannot be dissipated by viscous or capillary means any longer. The drop either deforms into an elongated liquid jet (Figure 1e) or atomizes [43] (Figure 1f). Jets are forming in the inertia dominant regime when $R_{d}\gg \lambda_{f}$ while atomization is significantly prominent in the capillary force dominant regime when $R_{d}\ll \lambda_{f}$. Here, $R_{d}$ denotes the characteristic size of the droplet while $\lambda_{f}$ represents the wavelength of the acoustic wave propagating within the droplet at frequency *f*. 

One can utilize the jet Weber number to make predictions about both the progression from the beginning of jetting to the formation of a single droplet as well as the later fragmentation of the jet into several droplets [47]:

(20) $${\mathrm{We}}_{j}\equiv \frac{\rho U_{j}^{2}R_{j}}{\gamma }$$

where $U_{j}$ is the velocity of the jet, $R_{j}$ is the jet radius, and γ is surface tension of the fluid. Tan et al. [47] have observed that the onset of jetting and the transition from single droplet pinch-off to multiple droplets formation occur universally at jet Weber numbers of 0.1 and 0.4, respectively, as a consequence of the axisymmetric breakup of the jet due to Plateau– Rayleigh instability. Further illustration is provided in Figure 1g.

### 2.3. Simulation of Droplet Ejection Using SAW

To better understand the internal mechanism of jetting induced by the interaction between SAW and droplets, finite element simulations are often employed for a more intuitive visualization. The deformation of the droplet interface is an exceedingly intricate phenomenon that encompasses various factors, comprising the interplay between sound waves and fluid, variations in surface tension at the gas-liquid interface, and the dynamics of the three-phase contact line (TPCL).

#### 2.3.1. Coupled Level Set with Volume of Fluid

Among existing methods to model interface flows, there are two commonly used Eulerian-based approaches including the volume of fluid (VOF) and level set (LS) methods. Although the VOF method maintains mass conservation, it suffers from low accuracy in catching sharp interfaces and curvature calculations in the context of surface tension as a dominant factor [56]. On the contrary, the LS method calculates the curvature accurately and captures sharp interfaces, but it often violates mass conservation [57]. The coupled level set with the volume of fluid (CLSVOF) method is capable of integrating the strengths of both the level set and volume of fluid approaches, enabling the attainment of a well-defined interface that maintains the mass of the entire system [54,58].

The VOF technique utilizes a VOF function denoted by the symbol *α* to describe the fraction of liquid volume present in a cell. The *α* value ranges from 0 to 1 in the interface region while it takes on values of 0 and 1 for the air and liquid phases, respectively. The transportation of the interface can be described by:

(21) $$\frac{\partial \alpha }{\partial t}+\nabla \cdot \left(u\alpha \right)=0$$

By introducing the liquid volume fraction *α*, the density ρ*,* and the viscosity μ of both the liquid and gas phases can be defined as:

(22) $$\rho =\rho_{l}\alpha +\rho_{g}\left(1-\alpha \right)$$

(23) $$\mu =\mu_{l}\alpha +\mu_{g}\left(1-\alpha \right)$$

Here, the subscripts ‘l’ and ‘g’ indicate the liquid and gas phases, respectively.

The volumetric surface tension $f_{\sigma }$ can be expressed as:

(24) $$f_{\sigma }=\sigma \kappa \left(\alpha \right)\nabla \alpha$$

where σ represents the surface tension coefficient of the liquid in the gas, and κ(α) represents the curvature of the interface, which can be calculated from:

(25) $$\kappa \left(\alpha \right)=-\nabla \cdot \frac{\nabla \alpha }{\left|\nabla \alpha \right|}$$

The accurate calculation of interface curvature is a challenging task due to the discontinuous nature of the step function definition of *α*, which is dependent on the gradient of *α* at the interface.

To achieve a sharp interface and smooth transition in physical properties across the interface, a coupled LS method with the VOF technique have been proposed. The approach involves introducing a LS field denoted as ϕ where the gas–liquid interface position is defined by the iso-surface ϕ=0. Notably, ϕ takes positive values in the liquid phase and negative values in the gas phase. The position of the interface is assumed to be at *α* = 0.5 at each computational fluid dynamics (CFD) time step; hence, the LS field is initialized as:

(26) $$\phi_{0}=\left(2\alpha -1\right)\cdot \Gamma$$

where Γ=0.75Δx, which is a small non-dimensional number whose value depends on the mesh size Δx. The initialized function $\phi_{0}$ is a sign function, which can be derived by solving the re-initialization equation:

(27) $$\frac{\partial \phi }{\partial \tau }=S\left(\phi_{0}\right)\left(1-\left|\nabla \phi \right|\right)$$

where $S\left(\phi_{0}\right)$ is the sign function of initial LS function, which is converged to a signed distance function when |∇ϕ|=0 while τ the artificial time step. Subsequently, the curvature at the interface and the surface tension force can be determined by:

(28) $$\kappa \left(\phi \right)=-\nabla \cdot \frac{\nabla \phi }{\left|\nabla \phi \right|}$$

(29) $$f_{\sigma }=\sigma \kappa \left(\phi \right)\delta \left(\phi \right)\nabla \phi$$

Through the utilization of the Dirac function δ(ϕ), $f_{\sigma }$ can be effectively localized to a restricted region in close proximity to the interface between two phases.

(30) $$\delta \left(\phi \right)=\{\begin{array}{cc} 0 & \left|\phi \right|>\epsilon \\ \frac{1}{2\epsilon }\left(1+\cos\left(\frac{\pi \phi }{\epsilon }\right)\right) & \left|\phi \right|\leq \epsilon \end{array}$$

where ϵ is the interface thickness that is chosen as ϵ=0.75Δx.

Analogously, the smooth Heaviside function H(ϕ) is employed to establish the density ρ and viscosity μ.

(31) $$\rho \left(\phi \right)=\rho_{g}+\left(\rho_{l}-\rho_{g}\right)H\left(\phi \right)$$

(32) $$\mu \left(\phi \right)=\mu_{g}+\left(\mu_{l}-\mu_{g}\right)H\left(\phi \right)$$

Here,

(33) $$H\left(\phi \right)=\{\begin{array}{cc} 0 & \mathrm{if}\;\;\phi <-\epsilon \\ \frac{1}{2}\left[1+\frac{\phi }{\epsilon }+\frac{1}{\pi }\sin\left(\frac{\pi \phi }{\epsilon }\right)\right] & \mathrm{if}\;\left|\phi \right|\leq \epsilon \\ 1 & \mathrm{if}\;\;\phi >\epsilon \end{array}$$

**Figure 2:**
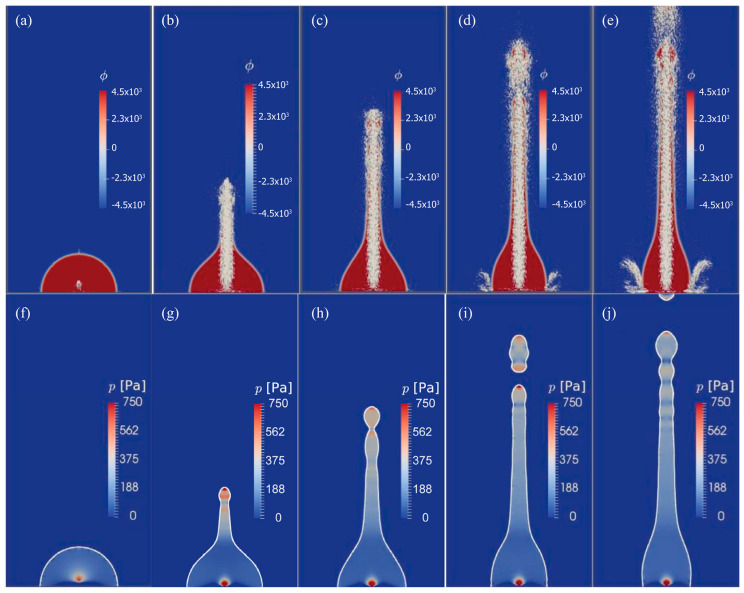
(**a**–**e**) Snapshots of the iso-surface α = 0.5 for droplet jetting phenomenon. (**f**–**j**) Snapshots of ϕ  overlaid by velocity vectors. Reprinted from Jangi et al. [59].

Jangi et al. [59] employed the CLSVOF method to simulate the liquid jetting induced by circular surface acoustic waves (CSAWs) on a ZnO film. Due to the in-plane isotropy of the ZnO film, sharp cylindrical liquid columns were generated by the ZnO/Si CSAWs device when subjected to acoustic energy from various directions within the plane. The following figures present the CLSVOF simulation results for the first 8 ms. Figure 2a–e displays the temporal evolution of the level set function ϕ, which can be applied to find out the evolution of the liquid–gas interface. Velocity vectors confirm that the jet is the result of a strong vertical momentum flow induced by circular focusing of the SAW. Figure 2f–j shows snapshots of the temporal variation in pressure distribution of the equal area α = 0.5 interface. The deformation of the droplet surface also drives the internal pressure, which is a function of the free surface curvature. High-pressure areas occur where the curvature at the jet tip is large while low-pressure areas occur where the interface curvature is reduced throughout the entire jet flow. These high- and low-pressure regions lead to local deformation and instability throughout the jet, resulting in the so-called Plateau–Rayleigh instability. 

Birouna et al. [54] conducted an investigation on the severe deformation/transportation/ejection behavior of sessile liquid droplets driven by surface acoustic wave devices, utilizing the CLSVOF method. Numerical results unveiled the fluid flow patterns and airflow velocity fields at various stages of droplet transportation/ejection. The impact of droplet volume, surface acoustic wave resonance frequency, and external surface acoustic wave power on droplet transportation/ejection was studied theoretically and experimentally. The model was able to predict the minimum radiofrequency power required to initiate droplet pumping and ejection at different resonance frequencies. 

Biroun et al. [60] investigated the jetting and internal flow behavior of liquid droplets using the CLSVOF method by analyzing the energy terms of the droplets in a liquid medium. Subsequently, a numerical model was employed to study the impact of the structure and location of two IDTs on droplet driving in order to achieve efficient mixing, separation, and jetting. The results indicated that two perfectly aligned IDTs were the optimal choice for mixing applications, whereas dual-offset IDTs were the best choice for concentration and separation applications. Using a pair of aligned IDTs, the maximum jetting velocity and shortest jetting time could be achieved while using two offset IDTs, effective liquid mixing and jetting could be observed.

#### 2.3.2. Lattice Boltzmann Method

In microscale systems, the intermolecular interactions determine the interface and dynamics of the contact line. Thus, mesoscale models, such as the lattice Boltzmann method (LBM), are more fit for simulating complex dynamic behavior without using level set, front tracking, or guessed interface shapes. Due to the mesoscale nature of this approach, it can make prediction about the motion of particles without considering slip boundary conditions, thus enabling simulation of moving contact lines. Tan et al. [61] utilized a mixed numerical scheme composed of finite difference technique for elastic solids and LBM for fluid flow to study acoustic streaming in micro- and nanochannels under surface acoustic wave excitation. The experimental results were consistent with the analytical results, indicating the feasibility of using the LBM to investigate liquid surface wave effects.

**Figure 3:**
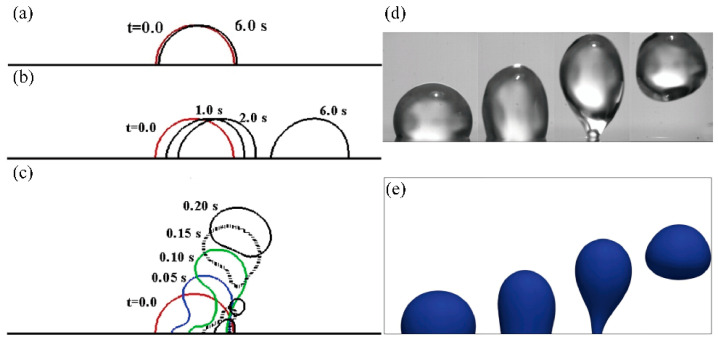
(**a**–**c**) 2D LBM simulation of dynamical behaviors of drop under SAW. Reprinted from Noori et al. [63] (**a**) streaming mode, (**b**) pumping mode, and (**c**) jetting mode. (**d**–**e**) A comparison between experimental and 3D LBM simulation of droplet jetting agitated by SAW. Reprinted from Burnside et al. [67].

Noori et al. [62,63,64,65] utilized a multiple-relaxation-time color-gradient model lattice Boltzmann method to establish a two-dimensional (2D) model for the interaction between droplets and SAWs (Figure 3a–c). By introducing a contact angle hysteresis model, the numerical accuracy was improved by 20% [62]. Combining LBM with experimental analysis of lithium niobate and zinc oxide-silicon devices, it was found that under the injection mode, a pair of oppositely rotating vortices was formed near the droplet, with stronger vortices observed on the zinc oxide-silicon device, resulting in a 70% reduction in droplet separation time [63]. Additionally, LBM simulation results showed that viscosity reduction, hydrophobicity, and surface tension coefficient increase significantly affected the flow and jetting control system and increased its sensitivity [64]. The dynamic behavior of droplets under microgravity conditions was affected by the Bond number with droplets able to move vertically on the surface when the Bond number exceeded a critical value [65]. Regarding drop removal using surface acoustic waves on a flat surface, LBM simulation results showed that the contact time was reduced by three times when a pair of SAWs with the same amplitude and frequency were applied [66].

For full capturing the underlying mechanisms and simulation of the droplet dynamics caused by internal flow induced by surface acoustic wave stimulation in three dimensions, Burnside et al. [67] established a three-dimensional two-phase lattice Boltzmann model and applied it to simulate the dynamic impact of SAW on micro-scale pinned droplets (Figure 3d,e). The model was validated by comparing the results of thermodynamic consistency, Laplace’s law, static contact angle on a plane, and droplet oscillation analysis with experimental data. The study found that the effects of forces, such as SAW, interfacial tension, inertia, and viscosity, on droplet mixing, pumping, and jetting dynamics were consistent with the observed and experimental data. The amplitude boundary of mixed, pumped, and jetted modes was established at different resonant frequencies. Additionally, how SAW substrate surface wettability, viscosity ratio, and interfacial tension influenced SAW-induced droplets were investigated.

### 2.4. Other Types of ADE

Currently, ADE systems based on focused acoustic transducer (FAT) can produce precise and uniform droplet ejection on demand, which is accomplished by focusing an acoustic beam on the liquid surface, creating sufficient pressure in a small region to overcome surface tension and expel a single droplet from an open space. In addition to SAW focusing transducers, which will be comprehensively discussed in the subsequent section, other types of FATs include concave transducers, self-focusing acoustic transducers, acoustic transducers with lenses, and acoustic transducers with resonator.

Concave acoustic transducers [68,69,70,71,72,73,74,75,76,77,78,79] can create focused acoustic radiation pressure with their unique concave design. Droplet diameter is proportional to focal spot size and inversely proportional to sound wave frequency. Optimal ejection can be achieved by adjusting distance between transducer and liquid surface and optimizing ultrasound pulse duration. Concave transducers are commonly used but challenging for higher frequency designs.

Self-focusing acoustic transducers [80,81,82] use a ring-shaped electrode design which called Fresnel half-wavelength band (FHWB) to create interference between sound waves for focusing. Droplet ejection direction depends on the size of inactive areas within the source region. Low viscosity fluids are preferred due to significant attenuation in a liquid pool. High viscosity fluids require more sources for higher sound pressure and narrower beams, but this makes manufacturing difficult.

Acoustic lens-based focusing [83,84,85] has a shorter focal length, enabling stimulation of smaller droplets. Through establish lens with air reflectors (LWAR), sound waves mainly reflect at the transducer-air interface due to impedance mismatch. A lens with an impedance between the liquid medium and the transducer, such as polystyrene, is used to make sound transmission efficient. When the polystyrene lens is made into a Fresnel half-wave zone, the sound waves interfere with each other to achieve focusing. The transducer with an air lens has smaller ejected droplet size and a more substantial focused sound pressure. Continuous large vibrations at the resonance point of the liquid-immersed air-backed transducer can cause structural fracture.

The utilization of various acoustic transducers with resonators has recently witnessed significant advancements. Foresti et al. [86] utilized the acoustic properties of a Fabry–Perot resonator to generate acoustophoretic force which enables the ejection of droplets ranging from micro- to nanoliter volumes. This approach allows for a variety of soft materials, including Newtonian fluids with viscosities exceeding four orders of magnitude (0.5 to 25,000 mPa·s) and yield stress fluids (*τ*_0_ > 50 Pa). He et al. [87] designed a gigahertz (GHz) acoustic resonator using CMOS technology. This acoustic resonator produces bulk forces at the solid–liquid interface, driving the liquid to produce sharp and stable “liquid needles”, then transporting the droplets to the target substrate surface via transient contact.

## 3. Influencing Factors of Surface Acoustic Wave Ejection

### 3.1. Factors of Fluid

#### 3.1.1. Volume

Alghane et al. [88] conducted experiments and found that the internal acoustic streaming effects of droplets reached their maximum when the characteristic diameter of the droplets R_d_/LSAWs ≤ 1, whereas a significant decrease in the internal acoustic streaming velocity was observed as R_d_/LSAWs ≥ 1. To achieve droplet ejection, the acoustic pressure needs to be sufficiently large to overcome the surface tension of the droplet’s free surface. When the droplet diameter is smaller than the attenuation length of LSAWs, LSAWs pass through the liquid/substrate interface, resulting in increased energy loss and decreased energy coupled into the droplet. As a result, the internal acoustic pressure within small-sized droplets is insufficient to observe the ejection phenomenon. The initial radius of droplets, which are initially stationary on the substrate surface, can be estimated using the following equation [38,89]:

(34) $$V=\frac{1}{3}\pi \left(3R_{d}-H\right)\times H^{2}$$

Here, $H=R_{d}\left(1-\cos\theta \right)$. Using this formula and assuming a volume of 1 μL and a static contact angle of 75°, the droplet radius R can be estimated to be approximately 0.7 mm. 

Wang et al. [90] presented experimental results on the jetting behavior of droplets under the influence of the propagating SAWs on an AlN device. The study demonstrates that mother droplet with size smaller than 1 μL break up and eject into smaller size droplets upon application of higher RF power to the device. Conversely, larger mother droplets tend to form cylindrical liquid beams, and an increase in droplet volume causes bending of the liquid at the front of the ejected beam due to gravity. The authors also report that the maximum jetting angles vary around 72.4° for droplets with volumes ranging from 0.2–10 μL. Wang et al. also investigated the jetting behavior of droplet under the influence of standing SAWs. The results show that the maximum jetting height of the droplet increases with a decrease in mother droplet volume in the range of 1–10 μL.

#### 3.1.2. Viscosity

The internal acoustic streaming in droplets arises from the dissipation of shear viscosities (μ) and bulk viscosities ($\mu_{B}$) in the liquid. As the viscosity and frequency increase, the internal flow becomes stronger while the radiation pressure is proportional to the energy reaching the free surface. Thus, higher viscosity leads to more dissipation and lower radiation pressure at the free surface [91], which in turn makes it more difficult to drive liquid movement and ejection. This is also evident from the attenuation length of sound waves in liquids, which indicates that:

(35) $$L=\frac{2\rho u^{3}}{{\left(2\pi f\right)}^{2}}\left(\frac{1}{\frac{4}{3}\mu +\mu_{B}}\right)$$

where *L* indicates the length of attenuation of the acoustic wave.

Bhattacharjee et al. [92] demonstrated that for low viscosity fluids, SAWs can be utilized to rapidly generate liquid bridges, which is seven times faster than current methods. In addition, with the reduction in the viscosity of the fluids, the duration for the liquid bridge to fragment into droplets also diminishes. Castro et al. [93] conducted an investigation on the formation of liquid bridges with varying viscosities ranging from 1.005 to 26.613 cP. They found that the increase in viscosity led to a decrease in the expansion and contraction of the impact film on the top plate. This outcome is similar to that of a decrease in the pulse width.

Li et al. [94] investigated the role of liquid viscosity plays in jetting behavior using propagating SAW on a ZnO/Si SAW device. The study demonstrates that increasing the liquid viscosity leads to an increase in the jetting angle and a decrease in jet velocity. Specifically, the jetting angle is 39° for DI water, but this value can be further increased up to 55° by increasing the kinematic viscosity. However, in liquids exhibiting greater viscous dissipation, the pressure distribution along the interface between the liquid and air might not generate enough force to counteract the surface tension force essential for initiating the formation of a jet. In addition, with the liquid viscosity increases, the average jet velocity U decreases. The relationship between the dimensionless velocity and the capillary number *Ca* can be expressed as:

(36) $$Ca=\frac{\mu U}{\gamma }$$

Pang et al. [95] have investigated the effect of capillary number on droplet deformation. They observed that viscous forces dominate the initial spherical droplet for large *Ca* values (i.e., *Ca* > 0.1). This caused the droplet to deform into an ellipsoidal shape before breaking into smaller droplets. Accordingly, the effect of viscous dissipation on the shape and angle of jetting beams is more pronounced with increased liquid viscosity in comparison to the role of surface tension. 

Burnside et al. [67] conducted LBM simulations to investigate the effect of different gas–liquid viscosity ratios (5, 15, 20) on jetting separation time. The research finds that an increase in the viscosity ratio leads to a proportional increase of the duration time for the droplet to expel from the substrate surface. This phenomenon can be explained by the deceleration of energy transmission caused by the viscosity’s increase of either the gas phase or the gas–liquid interface, consequently decelerating the movement of the interface along the Rayleigh angle.

#### 3.1.3. Surface Tension

The surface tension of a liquid tends to maintain the interfacial shape. However, the fluid will be ejected from the fluid–fluid interface when the inertial pressure in the fluid exceeds the surface tension. The jet Weber number, presented in Equation (20), is a well-known metric for this balance. In general, for smaller fluid geometries, the surface tension increases, and it is usually necessary to use a nozzle to generate sufficient fluid velocity to overcome surface tension. However, SAWs can produce very high surface acceleration and can couple with fluids to convert them into droplet velocity, thereby eliminating the need for a nozzle. Chen et al. [96] found that surface tension determines the maximum height of a droplet before its speed drops to zero as well as the volume of the ejected droplet. The sample liquids with varying surface tensions were prepared by adjusting the volume ratio of ethanol and water mixtures (the surface tensions of ethanol and water mixtures with volume ratios of 100% ethanol, 70%, 40%, 10%, and 0% water were 22.1, 25.5, 31.1, 49.5, or 72 mN m^−1^, respectively). Under a constant pulsed RF signal (pulse width of 780 μs and amplitude of 44 Vpp), the ejection height decreased from 2.82 mm to 1.48 mm when the surface tension increased from 22.1 mN m^−1^ to 72 mN m^−1^ in an inversely proportional function. The ejected droplet volume increased from 5 nl to 28 nl and showed a linear relationship with surface tension. The influence of surface tension on droplet shape is significant and can be described by the capillary number, *Ca*. At low capillary numbers, surface tension forces dominate, and the droplet remains spherical [95]. This is confirmed by Burnside et al.’s simulation [67] where droplets resist deformation due to SAWs at high surface tension and maintain a spherical shape. Decreasing surface tension allows the droplet to deform more along the Rayleigh angle, resulting in longer jetting. However, droplets are ejected from the substrate in approximately the same time under different surface tensions, and surface tension changes have a negligible effect on ejection time from the substrate.

### 3.2. Factors of Device

#### 3.2.1. Material Properties 

Piezoelectric materials, which can convert electrical signals into mechanical displacement via the inverse piezoelectric effect, have found widespread use in various mature industrial printing applications. Commonly used piezoelectric materials include piezoelectric single crystals, piezoelectric thin films, and piezoelectric ceramics, such as PZT. SAWs are mainly generated on piezoelectric single crystals (e.g., LiTaO_3_, LiNbO_3_) and piezoelectric thin films (e.g., ZnO, AlN). This section provides a brief introduction to these materials and their surface-treated properties.

- piezoelectric single crystals

**LiNbO_3_**: LiNbO_3_ crystal is highly attractive for microfluidic manipulation due to its high electromechanical coupling coefficient and low propagation loss, which enable Rayleigh SAWs excited on LiNbO_3_ crystal to strongly couple into the liquid along the propagation path. The propagation velocity of the SAWs in LiNbO_3_ crystal varies between 3400–4000 m/s depending on the tangential and propagation direction while the electromechanical coupling coefficient ranges from 0–5.6%. Among them, the most commonly used 128°Y-X LiNbO_3_ crystal exhibits the minimum parasitic signal level during propagation with an electromechanical coupling coefficient of 5.5% and a propagation velocity of 3980 m/s. Hereafter, unless otherwise specified, LiNbO_3_ crystal refers to 128°Y-X LiNbO_3_. As Rayleigh SAWs are composed of longitudinal and vertical polarized displacement components, tilted jetting along the Rayleigh angle can be generated on LiNbO_3_ crystal using a one-sided interdigitated transducer [9] or vertical jetting based on standing Rayleigh SAWs with two opposing focusing interdigitated transducers [97] or with circular interdigital transducers [98].

**LiTaO_3_**: The low coupling efficiency between SH-SAWs and fluids, which makes them unsuitable for fluid manipulation applications, has led to their predominant use in liquid sensing applications. However, Fu et al. [99] were able to induce droplet ejection along an almost vertical direction using a one-sided interdigitated transducer on a 36°Y-X LiTaO_3_ crystal. The aspect ratio of the ejected droplets was found to be proportional to the input power before the liquid column ruptured. This phenomenon was attributed to the gradual coupling of wave energy into the droplet via viscous effects of the liquid and the upward migration of wave pressure from the bottom, creating a nearly vertical pressure gradient within the droplet. This behavior is distinct from vertical jetting induced by LiNbO_3_ using two opposing interdigitated transducers.

- piezoelectric thin films

The use of thin film technology facilitates the integration of diverse functions onto a variety of substrates such as metal, glass, silicon, and polymer. Among these materials, polymers hold significant potential for the development of flexible and wearable devices [100]. Moreover, the thickness of the piezoelectric material used in the thin film affects the resonance frequency, thereby offering a means of tuning the device performance. Another advantage of thin film technology is the ability to selectively deposit the piezoelectric material onto regions where the acoustic wave is required [101].

**ZnO:** ZnO is commonly deposited on Si surfaces to produce ZnO/Si SAW devices. The velocity of the thin film ZnO surface acoustic wave is close to that of the Si substrate. With the thickness of the ZnO layer increases, the velocity gradually decreases and approaches that of bulk ZnO while the resonant frequency decreases. The ultra-smooth surface of the nanocrystalline diamond (UNCD) [95] thin film is beneficial for the formation of ZnO thin films with excellent c-axis orientation and low surface roughness. It also can suppress the attenuation of sound waves towards the substrate, making it very suitable for the fabrication of SAW devices. Due to the in-plane isotropic nature of the ZnO thin film, CSAWs can generate focused waveforms at the center of the device and induce uniform, slender, cylindrical jetting, without highly concentrated thin liquid columns observed in the case of anisotropic LiNbO_3_ using the same interdigitated transducer structure [59]. Fu et al. [102] characterized and observed Rayleigh mode SAW in ZnO/diamond SAW devices and achieved flow, pumping, and jetting of microdroplets with various sizes within different power conditions. Pang et al. [95] improved the liquid pumping speed and jetting performance of ZnO/Si devices by introducing a UNCD interlayer to form ZnO/UNCD/Si devices. Ong et al. [103] studied a transparent microfluidic device based on ZnO thin film/glass surface acoustic waves, verified its ability to transport, jet and atomize fluids, and applied it to active surface cleaning. ZnO can also be deposited on Al to form flexible ZnO/Al SAW devices with a jetting Rayleigh angle of 30.8° while it is 19.7° on ZnO/Si, and under the same input power, the lateral droplet transport of ZnO/Al is twice that of ZnO/Si [100].

**AlN:** Zhou et al. [104] employed magnetron sputtering to deposit aluminum nitride (AlN) thin films with various thicknesses (ranging from 2.3 to 4.7 µm) on a high-resistance silicon substrate. By utilizing AlN/Si SAW devices of different thicknesses, they achieved microfluidic operations, including flow, pumping, and jetting. The results demonstrated that the radio frequency (RF) power boundary required for transitioning from flow to pumping and from pumping to jetting decreased with increasing thickness of the AlN film. However, the flow velocity, pumping velocity, and device surface temperature all increased with increasing thickness of the AlN film. In addition to the Rayleigh wave mode, the AlN/Si SAW devices exhibited a high-frequency guided wave mode that could also be used for microfluidic manipulation [105]. Nevertheless, the low-frequency (Rayleigh) mode had a stronger microfluidic control effect. With the increase of RF power or the decrease of droplet size, both pumping and jetting velocities under the two modes increased. By changing the inclination angle of the AlN/Si device, tilted jetting was achievable. When the droplet volume was small, the jetting angles induced by a single IDT on both the flat and inclined surfaces followed the Rayleigh angle rule [90].

- Phononic crystals

**Figure 4:**
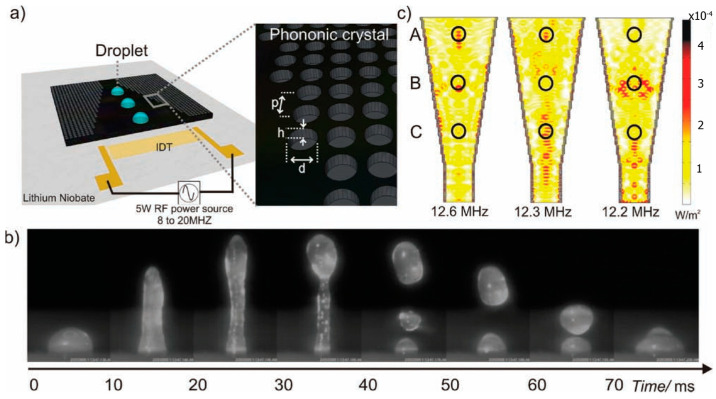
(**a**) The phononic crystal superstrate with the design of a conic structure deposited on the LiNbO_3_. (**b**) The jetting phenomenon induced by the acoustic waves on a phononic crystal superstrate. (**c**) simulation of the conic structure at three different input frequencies. Reprinted from Bourquin et al. [107].

Phononic crystals are a type of metamaterial consisting of a regular and ordered region with contrasting elastic moduli. Their application to microfluidic manipulation was first demonstrated by Wilson et al. [106]. Subsequently, Bourquin et al. [107] designed a phononic crystal structure (Figure 4) capable of regulating the position and intensity of acoustic energy on non-piezoelectric substrates and exploited the unique distribution of acoustic fields to achieve direction- and Rayleigh angle-independent jetting, resulting in high-amplitude and unstemmed droplet ejection at the liquid-air interface using only a single IDT.

- Hydrophobic treatment

**Figure 5:**
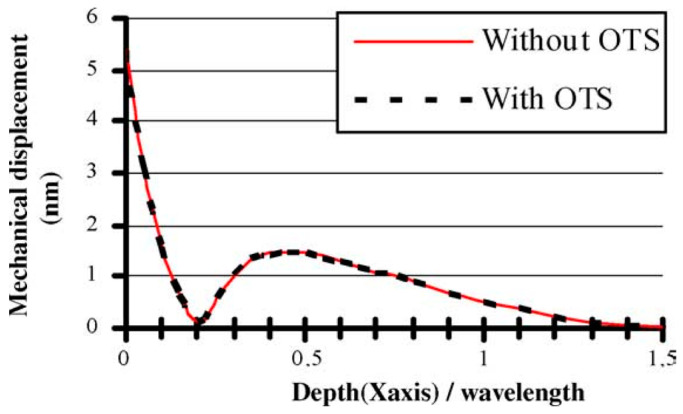
Vertical component of the sagittal-plane amplitude of the Rayleigh wave in LiNbO_3_ with/without OTS. Reprinted from Renaudin et al. [109].

On hydrophilic surfaces, the large interfacial tension hinders the energy transfer from SAW to droplets while hydrophobic surfaces are more favorable for droplet ejection. Deionized water typically exhibits hydrophilicity on common LiNbO_3_ substrates, with a contact angle of ∼75°. However, due to surface hysteresis, more energy is required to counteract surface forces for effective manipulation of droplets. The transport performance of droplets can be greatly improved by hydrophobizing the surface of the piezoelectric substrate [89,108,109]. Common surface hydrophobic treatments include coating the surface with a Teflon^®^ layer or surface silanization [110,111,112]. The contact angle of the treated surface can reach ~105°. Silanization treatment forms a Self-assembled monolayer (SAMs) on the surface, which is composed of molecules with a strong and specific affinity for a particular surface composition. Commonly used self-assembled organic silanes for microfluidic manipulation or on-chip laboratory include OTS, OTMS, FTDS, etc. As illustrated in Figure 5. The influence of the OTS monolayer on the SAW propagation is negligible [109,110].

**Figure 6:**
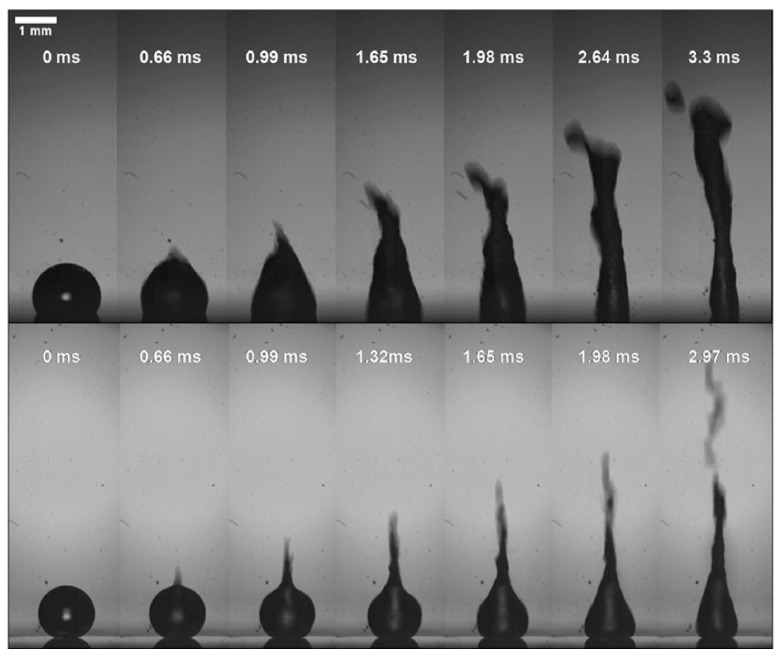
The image sequence in the top row depicts the deformation of droplet jetting on a hydrophobic substrate coated with a Teflon film that is approximately 300 nm thick. In contrast, the bottom row illustrates the formation of jetting on a superhydrophobic substrate that has been treated using the plasma coating method. Reprinted from Darmawan et al. [115].

Regarding droplet ejection, Shiokawa et al. [9] first found that when stearyltrichlorosilane was applied to construct a chemically hydrophobic surface, a stream of droplets was ejected from the liquid upward at a Rayleigh angle. The power required to eject droplets to the same distance will be reduced by about 40% through hydrophobic treatment [113]. At the same time, the number of satellite droplets will be significantly reduced. To achieve better ejection performance, Tan et al. and Bhattacharjee et al. [47,92] coated the substrate with a thin layer of Teflon while Bussonniere et al. [114] used OTS treatment. As the surface wettability increases, such as on a superhydrophobic surface, the droplet ejection behavior will change significantly. Darmawan et al. [115] obtained a superhydrophobic substrate with a water contact angle of about 155° by direct plasma treatment of He, CH_4_, and C_4_F_8_ gas mixtures. Compared with the substrate coated with 300 nm thick Teflon (contact angle of about 105°), a sharp ejection pinch was formed at the tip of the droplet instantaneously (for instance, the image at 0.66 ms in Figure 6) instead of at the droplet pinning point. Since the inertial force is mainly concentrated at the tip of the droplet, the ejection speed is faster, the jet radius is smaller, and the jet shape gradually changes to multiple jets. This is mainly due to the smaller effective surface area of the superhydrophobic substrate for efficient surface acoustic wave propagation, which leads to a shorter wave propagation path. The surface wave that leaked out was mainly constrained to the restricted point where the liquid made contact with the substrate and advanced along a relatively concentrated path leading straight up to the highest point of the droplet [115].

#### 3.2.2. Type of Interdigitated Transducers 

**Figure 7:**
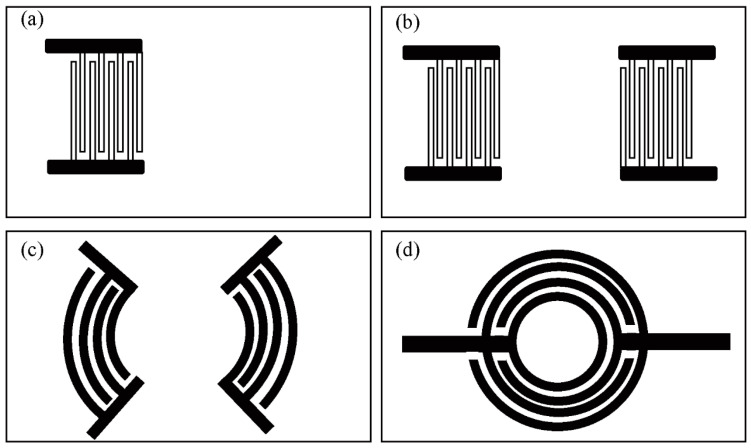
(**a**) Single straight IDT. (**b**) Double straight IDTs. (**c**) F-IDTs. (**d**) C-IDTs.

In R-SAW devices, single-port IDT (Figure 7a) typically induce droplet ejection along the direction of the Rayleigh angle. However, it is also possible to achieve vertical jetting by utilizing higher-order waves, such as LiNbO_3_ [116] or SH-SAW in LiTaO_3_ [99]. Lamb waves have long been considered less efficient than SAWs for microfluidic driving, with an efficiency difference of approximately one order of magnitude [117]. Nevertheless, driving droplet atomization and jetting via the anti-symmetric A0 mode of Lamb waves can still be achieved by employing a double-sided single-phase unidirectional transducer (SPUDT) and implementing an optimized design to enhance the A0 mode and suppress the S0 mode [118]. Excluding the aforementioned IDT designs, in order to achieve vertical injection, IDTs are commonly designed as circular (Figure 7d), elliptical focusing (Figure 7c), and double straight (Figure 7b) configurations. These specific IDT designs have been widely utilized and proven effective in facilitating vertical injection.

- Circular interdigital transducers

Circular interdigital transducers (C-IDTs) have a significant advantage in that they focus acoustic energy at the center of the ring, enabling vertical droplet injection. Demirci et al. [119,120,121] established a two-dimensional droplet jetting array using C-IDTs and applied it to ultra-high-throughput single-cell jetting. Zahertar et al. [98] deposited standard C-IDTs on a 128° Y-cut LiNbO_3_ substrate, which allowed for acoustic energy from all directions to converge on the central focal point. They demonstrated the driver’s acoustic flow manipulation ability by jetting DI water droplets at 5 W. The pulse width modulation of the C-IDT control signal allows for precise control of droplet generation time and sequence, as well as accurate droplet volume [96]. The anisotropy of LiNbO_3_ crystal leads to the formation of liquid columns that resemble concentrated thin liquid columns upon jetting. In contrast, ZnO thin film is an isotropic material. Jangi et al. [59] have used C-ITDs to induce the formation of uniform, slender, cylindrical liquid columns on the surface of ZnO that are distinct from those formed on LiNbO_3_.

Multi-IDT arrays, such as the eight transducers arranged as an octagon array, have been widely used in cell manipulation due to their highly flexible phase modulation capability [27]. This array has a structure similar to that of C-IDTs, and it focuses the Lamb wave acoustic focusing area on a single vertical line at the center. By combining the mechanisms of acoustic pressure waves and acoustic flow, jetting in a large working range of liquid height can be achieved [122].

- Elliptical focusing interdigital transducers

Compared to conventional SAW devices with similar substrate dimensions and IDT periods, the F-SAW device, equipped with elliptical focusing interdigital transducers (F-IDTs), has been found to be capable of exciting high-intensity and high-beam-width compressed waves in a small focal range. The focal region is sensitive to changes in amplitude, and the maximum flow force at the focal point increases by an order of magnitude with the square of the applied input voltage, which is higher than that of existing conventional SAW devices. The F-SAW device has higher insertion loss, making it more suitable as a driver than a sensor [50]. Tan et al. [47] first used standing Rayleigh SAW generated from two opposite F-IDTs to achieve vertical ejection and found that droplets larger than the acoustic wavelength of the fluid form elongated jets above the critical Weber number while Connacher et al. [123] used F-IDTs to achieve nozzle-less controlled ejection of droplets at fixed angles. This controlled angle implementation is due to the different control signals of the two pairs of IDTs and the basic parameters of the initial fluid.

- Double straight interdigital transducers

C-IDTs and F-IDTs can focus acoustic energy on a single point or a small region, eliminating the need to consider the IDT aperture. In contrast, double straight IDTs belong to the non-focusing type, and SAWs propagate within the aperture width range of the IDTs. The aperture length of the IDT refers to the overlapping region of the interdigitated electrodes while the aperture width determines the width of the SAW beam. The acoustic energy is uniformly distributed within the aperture range between the two pairs of IDTs, and a larger aperture size leads to a larger excited signal and a corresponding lower energy consumption. However, an excessively large aperture results in a larger parasitic capacitance, which is not conducive to high-frequency signal transmission. On the other hand, a too small aperture is also disadvantageous for the device, as it enhances SAW diffraction effects. Therefore, to achieve optimal ejection, the aperture of the double straight IDTs should be adjusted within a suitable range. 

By exciting two opposite columns of SAWs simultaneously through the double straight IDTs, energy leakage into a liquid droplet can be controlled to achieve controlled droplet ejection in a desired direction. By varying input RF power to the two symmetric IDTs or tuning the energy of two opposite SAWs, the direction of ejection can be manipulated. When the two opposite SAWs share the same energy, the expulsion of the droplet would occur at an ideal 90-degree angle relative to the piezoelectric substrate due to two opposite and equal SAW streaming forces from both sides [18]. Yet when the two opposite SAWs do not share the equivalent energy, deviation from the vertical axis will occur when the droplet ejects and the droplets will turn to the side which has smaller energy distribution [93,94].

#### 3.2.3. Types of Waves Excitation

**Figure 8:**
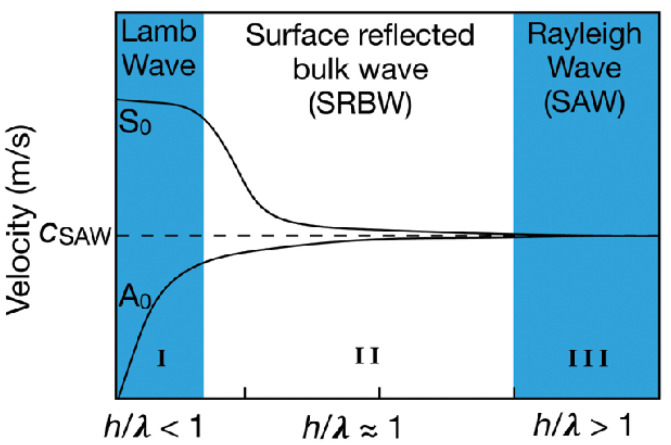
Sketch of the wave dispersion curves showing the fundamental antisymmetric (*A*_0_) and symmetric (*S*_0_) modes. Reprinted from Rezk et al. [117].

The penetration depth of surface acoustic waves (SAWs) is inversely proportional to their resonant frequency *f* [9].To prevent leakage and reflection of SAWs through the substrate, conventional substrate thickness *h* is designed to be much greater than the wavelength *λ* of the SAWs. That is to say, the vibrational energy of SAWs is confined to a region along the substrate surface with a characteristic thickness, typically on the order of λ_SAW_ = c_SAW_/*f*, where c_SAW_ is the Rayleigh phase velocity of the SAWs [117]. Therefore, by adjusting the ratio of SAW wavelength *λ* to substrate thickness *h*, different types of acoustic waves can be excited (Figure 8).

- Lamb waves

When the ratio of substrate thickness to SAW wavelength, *h*/*λ* < 1, Lamb waves are excited in the substrate. However, Lamb waves have weaker fluid driving capabilities compared to Rayleigh AW, so reasonable optimization of the IDT is necessary to enhance the driving capability. Ning et al. [122] focused the Lamb waves using eight transducers arranged in an octagonal array and achieved a continuous injection rate of 0.33 kHz of uniform water droplets with a diameter of 22 μm and a volume of 5.6 pL. Fu et al. [118] proposed an optimized double-sided SPUDT to enhance the anti-symmetric A0 mode of Lamb waves and fabricated a Lamb wave device on a double-sided polished 128°YX LiNbO_3_ silicon wafer with a frequency of 7.9 MHz, verifying the feasibility of injection and atomization.

- surface reflection body waves

When *h*/*λ* ≈ 1, surface reflection body waves (SRBWs) [117] generated by Hybrid Resonant Acoustic (HYDRA) appear on both sides of the substrate with IDTs on one side and their corresponding bottom surfaces. SRBWs have the advantages of both surface waves and body waves, making them applicable for fluid manipulation and sensing [124,125]. Rezk et al. [126] integrated the HYDRA platform at the bottom of a 96-well plate, achieving a mixing, concentrating, and injection platform with single or multiple well addressing functions. By triggering SRBW pulses under each well, multiple wells can be synchronously or asynchronously continuously injected, and the droplet size is influenced by the input power.

- Rayleigh SAW

When *h*/*λ* > 1 (or more strictly, *h*/*λ* > 3), the device excites the conventional Rayleigh SAW. Rayleigh Surface Acoustic Waves (SAWs) are characterized by the elliptical trajectory of the surface particles of an elastic body near its surface. The energy of Rayleigh SAWs is mainly concentrated within 1~2 wavelengths of the surface. When Rayleigh SAWs propagate on the semi-infinite elastic body surface, the displacement of the surface particles can be decomposed into two directions: along the propagation (horizontal) direction of the SAWs and perpendicular to the elastic substrate. Rayleigh SAWs are non-dispersive waves whose velocity depends only on the material parameters of the propagating medium and is independent of frequency. Due to their strong coupling with fluids, Rayleigh SAWs have been widely used in microfluidic manipulation. When using Rayleigh SAWs to excite droplet jetting, the droplet generally jets out along the Rayleigh angle due to the difference in sound wave propagation speed between the substrate and the fluid. To achieve vertical jetting, multiple directions of Rayleigh SAWs can be combined to cancel out the horizontal component and enhance the vertical component. This can be achieved by using symmetric IDTs, such as two opposite IDTs, octagon array IDTs, and circular IDTs.

- High-order wave

**Figure 9:**
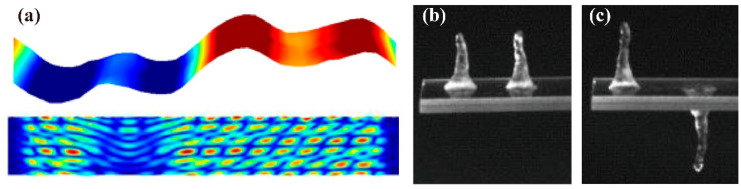
(**a**) The vibration characterization of acoustic wave measured by laser Doppler vibrometer (LDV). (**b**) Droplet array jetting phenomenon induced by high-order modal acoustic wave both on the upper surface (**c**) or on the upper and lower surface at the same time. Reprinted from Lei et al. [116].

Lei et al. [116] reported that LiNbO_3_ can support higher-order mode acoustic waves when excited at frequencies beyond the conventional Rayleigh SAW excitation frequency. As the ratio of the piezoelectric substrate thickness to the Rayleigh wavelength increases, the bandwidth of the higher-order mode acoustic waves becomes narrower, leading to a less pronounced effect. This effect may derive from the reflection wave from the bottom of the piezoelectric substrate and the second-order effect of IDTs. By utilizing the high-order wave excitation for jetting, it is possible to achieve vertical jetting on both sides of a double-sided polished piezoelectric substrate using only a single IDT (Figure 9b,c). This is in contrast to traditional Rayleigh SAW.

- SH-SAW

There is limited research on SH-SAW induced ejection, and more information can be found in the “piezoelectric single crystals” section regarding LiTaO_3_.

### 3.3. Presence of Channel or Chamber

#### 3.3.1. Sessile Droplet

A sessile droplet is a liquid droplet placed on a substrate without any additional confinement.

- Jetting

When a SAW propagates across the substrate and contacts the droplet, a portion of the LSAW energy couples into the droplet. When the energy of the LSAW is sufficient, the droplet will form a slender jet along the Rayleigh angle. To achieve vertical jetting, symmetric IDTs can be applied with the droplet placed in the middle of the IDTs, and equal power is applied to both sides. Shiokawa et al. [9] first conducted a sessile drop jetting experiment utilizing a single IDT and found that R-SAW can drive the liquid to eject. Although SAWs have been used for driving sessile droplets later, it was not until 2009 that Tan et al. [97] systematically explored how jet is formed through a pair of elliptical focusing EWC-SPUDTs and summarized the effects of Reynolds (Equation (19)) and jet Weber numbers (Equation (20)) on jetting. Bourquin et al. [107] used a single IDT to induce vertical jetting of sessile droplets through the integration of phononic crystals into SAW devices. Since then, various materials, such as ZnO [95,102] and AlN [104,105], have been used to drive and eject sessile droplets. In 2017, Fu et al. [99] used LiTaO_3_ to generate shear-horizontal SAW and achieved vertical jetting of sessile droplets using a single IDT. Later, the use of various types of SAW to drive sessile droplet ejection has emerged. Recently, Lamb waves [122] and high-order waves [116] have successfully enabled jetting of sessile droplets.

- Liquid Bridge

**Figure 10:**
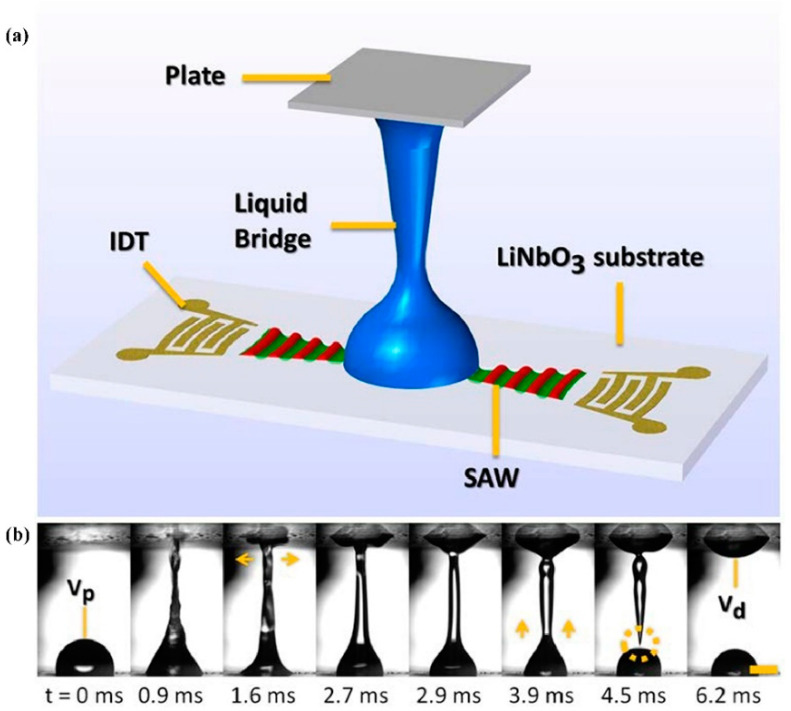
(**a**) Schematic illustration of the formation of a liquid bridge induced by SAW. (**b**) The deformation of the liquid bridge captured by the time series of images obtained via high speed video. V_p_ refers to the volume of the mother droplet while V_d_ refers to the volume of the ejection droplet. Reprinted from Castro et al. [93].

In general, a liquid bridge or a capillary bridge is a liquid residing between two solid surfaces separated by a certain distance through the action of surface tension [127]. Surface acoustic waves (SAW) generate a driving force of up to an order of 10 million *g* on a piezoelectric substrate, which can be used to eject liquid samples to a desired location and form a liquid bridge [93] (Figure 10). In 2010, Bertin et al. [128] formed a liquid column with an aspect ratio exceeding the Plateau–Rayleigh instability by injecting an acoustic beam into the top of a cylindrical cavity using SAW. As low-viscosity liquid samples have weaker surface tension, the stretching method commonly used for bridge formation is time-consuming and prone to rupture. By using a pulsed surface acoustic wave, a capillary bridge can be formed within several milliseconds from a stationary low-viscosity liquid droplet [92], facilitating the investigation of its capillary thinning behavior and measurement of the fluid’s extensional viscosity. The liquid bridge can also be used for the distribution of trace amounts of samples. Castro et al. [93] utilized a SAW liquid bridge to deposit samples with precise and repeatable volumes onto a target substrate within 10 ms with a minimum volume of 0.22 μL, a standard error of 6.5%, and a coefficient of variation of 11.3% [93]. By applying unmatched input energy to the interdigital transducer, multi-point droplet directional ejection can also be achieved, greatly improving the distribution efficiency without the need to reposition the chip between distribution steps.

- Pendant

**Figure 11:**
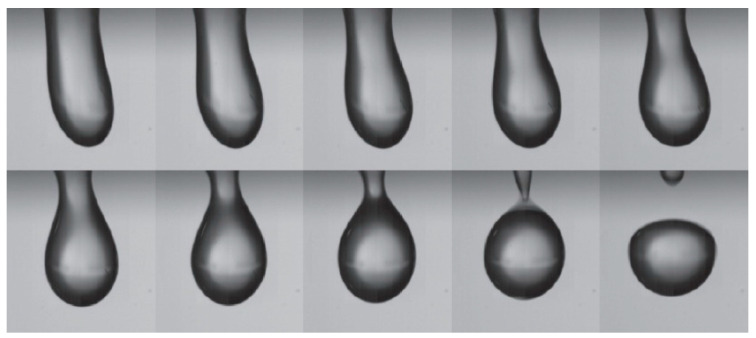
Sequence of images (time elapsed between successive images: 2 ms, captured at 500 fps) showing the detachment of a drop of 10 μL excited with SAW. Reprinted from Bussonniere et al. [114].

In the pendant state, droplets are directly affected by gravity. The force even strongly influences the small droplets’ dynamics, and a quasi-static equilibrium appears as the droplet approaches the separation threshold. By comparing the pendant droplets and dynamics of sessile, the influence of gravity on the oscillation frequency *f* and amplitude *h* of the droplets can be determined. As droplets exhibit nonlinear oscillation, their particular frequency depends on the average stretching of the droplet shape. For pendant droplets, radiation pressure and the static effects of gravity mainly induce this stretching in the same direction. In contrast, for sessile droplets, radiation pressure and gravity are opposed, and it is the nonlinear dynamical effects that mainly induce the average stretching, which depend on *h*. In this case, *f* is strongly influenced by *h* [114]. Figure 11 showed the detachment of a pendant drop of 10 μL excited with SAW.

#### 3.3.2. Channel

- Droplet production or splitting in the channel

The use of SAWs to drive the formation and manipulation of liquid droplets has become an effective tool in droplet generation, splitting, and production. This is achieved by utilizing the ARF generated at the interface between two fluids with different acoustic impedances, which creates a density gradient that induces the deformation of the liquid-liquid interface and the formation of dispersed phase droplets in the continuous phase. 

**Figure 12:**
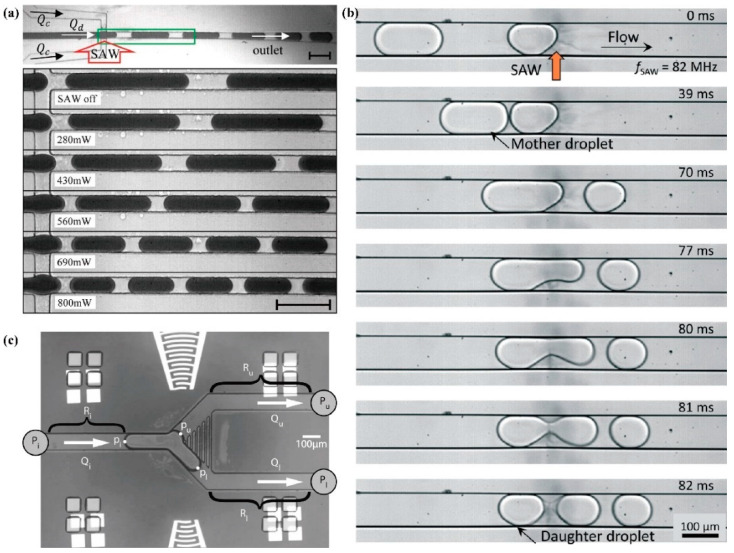
(**a**) Effect of the SAW on droplet formation and size, the thin black arrows indicate the flow directions, while the thick red arrow indicates the direction of the SAW. Reprinted from Bussonniere et al. [129] (**b**) Droplets in oil can be split into two or more differently sized daughter droplets utilized the SF-IDT, the orange arrow indicates the direction of the SAW. Reprinted from Jung et al. [131] (**c**) The generation of liquid droplets in the Y-junction is achieved using F-IDTs, Q denotes flow rates. Reprinted from Sesen et al. [132].

In vertically intersecting microfluidic channels, the size of the droplets can be controlled by the SAW power [129] (Figure 12a). In T-junction channels, Schmid et al. [130] demonstrated real-time adjustment of droplet size under low capillary numbers using unfocused SAW. In a system integrated with a slanted-finger interdigital transducer (SF-IDT), droplets in oil can be split into two or more differently sized daughter droplets by adjusting the input voltage and flow rate [131] (Figure 12b). Similarly, in a Y-shaped microfluidic chip integrated with a F-IDTs, precise control of the acoustic radiation pressure applied to the oil–water interface can guide the droplets into one outlet or another or split them into desired proportions [132]. SAW can also be used to selectively inject (or extract) part of a droplet from a moving droplet into a customized reaction chamber. Sesen et al. [133] achieved accurate operation of droplets in the range of 150 pL to 850 pL using a SAW pipette (PoaCH) system (Figure 12c). The irreversibly bonded piezoelectric substrate and microfluidic chip limits the flexibility of on-chip droplet splitting. Park et al. [134] eliminated the need for permanent bonding by using a disposable polydimethylsiloxane microfluidic chip and demonstrated the feasibility of droplet tri-splitting using parallel-type SAW-based acoustofluidics. Mutafopulos et al. [135] generated droplets in microfluidic devices using traveling-wave surface acoustic waves (TSAWs) and combined droplet generation with fluorescence detection to selectively encapsulate cells and microspheres. In 2020, Park et al. [136] used F-IDT excited SAW in a channel to achieve acoustic streaming effect, and obtained a flow with precise chemical concentration, which was then introduced into a T-junction to generate droplets with adjustable chemical concentration. In summary, by utilizing the ARF generated by SAWs on the oil-water interface, droplets with controllable volume, number, and internal concentration can be generated as needed. Pulse duration determines the number of droplets while SAW power, continuous phase flow rate, pulse duration, and channel geometry all influence the droplet size [137,138]. 

In addition to using SAW splitting to form droplets, Sesen et al. [139] used surface acoustic waves to induce ARF to fix continuously pumped small droplets in an expansion chamber to form aggregates with consistent sizes until they reached a critical size, at which point the drag force of the flowing oil phase overcame the trapping force, allowing the aggregates to leave the chamber.

- Jetting in the chamber

Acoustic droplet ejection is often used with a chamber installed on a focused acoustic transducer (FAT) as a liquid storage device. However, due to the relatively weak energy of SAW compared to other ultrasonic methods, the use of storage tanks requires caution, and there are differences in the placement and structural design. 

**Figure 13:**
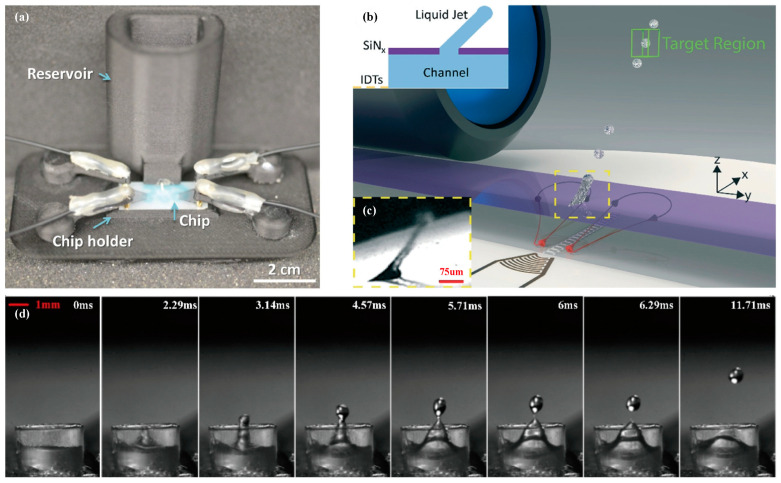
(**a**) The SAW jetting system integrates a liquid reservoir and self-feed (spontaneous capillary imbibition with a pulled capillary tube) together with the piezoelectric chip. Reprinted from Castro et al. [140] (**b**) Droplet ejection from the teardrop shaped nozzle on the SiN_x_ membrane. (**c**) The highspeed imagery of droplet ejection through tear drop shaped nozzle. Reprinted from Brenker et al. [141] (**d**) The evolution of single droplet jetting and pinching-off in a vertical capillary. Reprinted from Lei et al. [142].

Castro et al. [140] placed a liquid reservoir outside of the SAW chip and drove self-supplied liquid through a capillary into the focus position of the SAW to continuously produce single droplets with diameters ranging from 60 to 500 microns (Figure 13a). The droplet size was flexibly adjusted by the duration of the pulse width, which avoided the need for separate devices when using nozzles or frequency control for different droplet sizes. 

Brenker et al. [141] proposed a teardrop-shaped nozzle on a closed chamber to achieve liquid droplet ejection into the air (Figure 13b,c). By combining acoustic radiation force and fluid flow, the precise interval control of 40 μm droplets with a duration of 61 μs was achieved, and the droplets were directed through a 500 μm square target area. The synchronization frequency of the ejection to an external source was adjusted by modifying the pulse length, reaching up to 357 Hz, which exceeds the repetition rate of all currently operating XFELs (X-ray free-electron lasers) worldwide.

When mounting vertical capillaries on a surface acoustic wave (SAW) device, the SAW injection capacity can be enhanced, leading to more standardized and controllable injection methods (Figure 13d). However, this also increases device complexity and requires more power to maintain injection due to the capillaries’ absorption of SAW energy and increased liquid volume. Lei et al. [142] have pointed out that to achieve good injection effects, it is necessary to maintain a stable liquid surface inside the capillary, with the inner diameter controlled between 2–4 times the SAW attenuation length. The optimal fluid position is above *h* = *D*/2tan*θ_R_* and below the capillary’s force burst threshold, where *θ_R_* is the injection tilt angle and *D* is the capillary inner diameter. As the liquid level increases, the droplet size decreases inversely.

Chen et al. [96] proposed a method for generating liquid droplets without the need for auxiliary oil by bonding a PDMS circular storage device on top of a C-IDT. The method uses a modulated focused SAW to induce deformation at the liquid–liquid interface, generating droplets on demand. The authors demonstrated the capability of the technique by generating droplets with a volume of 24 picoliters within 10 milliseconds and encapsulating single cells in the droplets. The suggested approach presents a potential substitute for droplet production in microfluidic channels without the requirement of oil, making it a highly favorable alternative for droplet generation. Moreover, it represents a commonly adopted strategy for ADE using Surface Acoustic Waves in biochemical analysis applications [143,144,145,146,147,148,149,150,151].

### 3.4. Factors of Driving Signal

#### 3.4.1. Input Power

Increasing the input power directly results in the amplification of the substrate vibration amplitude. The amplification can be confirmed by measuring the vibration amplitude within the nanometer range using an optical method, which is proportional to the RF power [152] as described by Equation (18) at a constant wavelength. When the vibration amplitude is relatively small, stable acoustic streaming occurs in the liquid, and the acoustic streaming velocity increases with increasing power. This is due to the increased momentum transferred to the liquid in the form of a jet, resulting in significant inertial effects that lead to chaotic mixing. The SAW streaming force is directly proportional to the square of the SAW amplitude, as shown in Equation (17). When the amplitude exceeds a certain threshold, the droplet will begin to move, and its speed increases with increasing amplitude. After a sharp increase, the speed saturates at a volume-dependent value [91]. As the power further increases, jetting occurs when the energy of the liquid is sufficient to overcome the surface tension. The height and speed of the jet increase with increasing power, and multiple droplet ejections may occur when the jet Weber number (as described by Equation (18)) is greater than 0.4 [47]. Rezk et al. [126] demonstrated that the size of the ejected droplets can easily be adjusted by altering the input RF power. With an initial liquid volume of 200 μL and a pulse duration of 7 ms, the ejected droplet size reduced from approximately 730 μm to 320 μm with an increase in input power from 2.95 W to 3.55 W. Castro et al. [93] demonstrated that the volume of ejected droplets, normalized against the initial parent drop volume, as a function of the applied power. Their study further revealed that by modulating pulse duration and the input RF power, the ejected droplet volume of 50% DI water/glycerol liquid can be tuned from roughly 35% to 70% of the original parent drop volume. 

#### 3.4.2. Frequency

Under the same input power condition, the amplitude and energy of surface acoustic waves decrease with increasing frequency due to the higher energy dissipation rate at higher working frequencies. This is reflected in the inverse proportionality of the attenuation length to the square of the frequency, as shown in Equation (35), which is independent of the applied power. Increasing the device frequency shortens the interaction distance between SAWs and liquids, leading to stronger energy absorption and dissipation by the liquid. As a result, a higher input power is needed to achieve effects similar to those at lower frequencies. Dentry et al. [49] studied the effect of applied frequency on SAW-induced streaming and found that SAW energy leaks into the liquid, producing a jet flow, and the flow velocity decreases as the jet moves away from the sound source due to viscous dissipation. The length of the jet is determined by the attenuation length of the SAW, which decreases with increasing frequency, leading to an increase in power density and faster jet velocity. The velocity reaches a maximum at a certain position along the propagation axis before decreasing again. Guo et al. [153] observed jetting in a 5 μL water droplet on bi-directional SAW devices with different resonant frequencies (61.7 MHz, 110.8 MHz, 199.4 MHz, and 250.1 MHz). They found that higher resonant frequencies require higher power thresholds for jetting due to greater energy absorption rates at high frequencies. This is consistent with Equation (1), which indicates that the attenuation coefficient is proportional to the frequency, suggesting that attenuation increases with SAW frequency. 

#### 3.4.3. Input Signal Modulation

Through modulation of the input signal, power supply requirements can be reduced for achieving the same jetting performance, which in turn can minimize substrate heating effects and enhance the thermal stability of the control system. Shiokawa et al. [52] demonstrated jetting formation in a 6 μL droplet excited by the input interdigital transducer at a frequency of 50 MHz with a pulse frequency of 100 Hz and SAW input power of 2 W. Yet a high-quality fog was generated when the pulse frequency is 1 kHz. Bennes et al. [113] measured the ejection distance: when the pulse duration is short (to be precise, below 50 ms), the ejection distance of the droplet rises along with pulse duration while when the pulse duration is equal to or larger than 50 ms, the ejection distance of the droplet appears to remain the same, and atomization can be observed when the pulse duration is higher than 1 s. Baudoin et al. [154] proved that by modulating the input signal to around one to two times the Rayleigh–Lamb characteristic frequency, the acoustic power required for SAW stirring, stretching, or moving a sessile droplet can be reduced. Specifically, the minimum power needed to vertically stretch the droplet by 0.2 and 1 times was reduced by 5 and 3 times, respectively. Castro et al. [140] demonstrated the production of single or multiple droplets with diameters ranging from 60 to 500 μm by flexibly adjusting the pulse width and duration without the need for different device configurations for different droplet sizes.

### 3.5. Summary

Although there are various substrate materials, such as piezoelectric films, that can be utilized for droplet ejection, the piezoelectric coupling coefficient of piezoelectric films is relatively small. Furthermore, the elasticity, temperature characteristics, and dielectric properties of the film can affect its piezoelectric properties. Moreover, even with different materials, there may be some differences in experiments due to different processing techniques or differences in process accuracy. In addition, the power used for piezoelectric single crystals is smaller than that used for piezoelectric films, and the thermal effects on the substrate are weaker. Among different excitation waveforms, such as R-SAW, Lamb wave, SH-SAW, and High-order wave, R-SAW exhibits the best coupling performance with fluids, making LiNbO_3_ the most suitable substrate for microfluidic ejection in general applications.

For applications that require vertical ejection, using a F-IDT can achieve more prominent ejection performance. However, to achieve this effect, the droplets need to be accurately placed at the acoustic focal point. Otherwise, the liquid will be ejected in different directions due to the different energy distribution in different regions, making materials such as LiTaO_3_ or High-order waveforms more meaningful as they can achieve vertical ejection without considering the placement of droplets.

By introducing microfluidic channels, SAW can be integrated into Lab on chip and play a significant role in biochemical analysis/reaction. Assembling the chamber can also improve the storage performance of SAW devices, thereby enhancing ejection efficiency. However, as Castro et al. [12] concluded, the stability of ejection imposes strict requirements on chamber size, the height, and smoothness of the liquid in the chamber, which poses a challenge to the widespread application of SAW. Although the storage tank and capillary used by YEO et al. [140] do not have such problems, the limitation of capillary effects makes it difficult to achieve high frequencies. Therefore, discovering how to ensure the stability and uniformity of ejection while increasing the ejection frequency is also a challenging issue.

Higher driving power will produce a large number of satellite droplets or even atomization, which is disadvantageous for on-demand single droplet formation. However, too low power cannot produce ejection or even cause breakage. Therefore, the ejection power needs to be optimally designed by combining liquid parameters and material characteristics. Modulated triggering signals show superior performance in fluid manipulation and atomization compared to continuous excitation. It can not only reduce the thermal effects on the substrate while lowering the total input power, but also enhance the stability of manipulation. However, its effect on droplet ejection performance and its inherent mechanism have not been reported.

## 4. Application in Bioprinters and Chemical Analysis

### 4.1. Encapsulation of Circulating Tumor Cell

**Figure 14:**
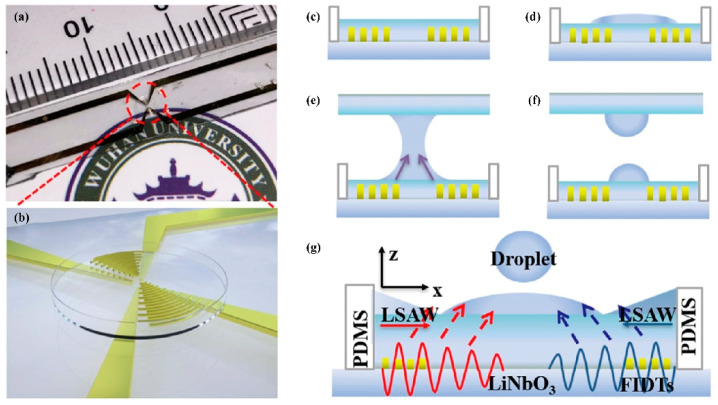
(**a**) shows a photograph of the device utilizing a LiNbO_3_ substrate coated with a F-IDT and a circular reservoir. (**b**) presents a schematic enlargement of the reservoir and F-IDT. (**c**–**g**) illustrate the droplet generation mechanism using the FIDT. Reprinted from Li et al. [144].

The recovery of single circulating tumor cell (CTC) of patients offers possibilities for promoting research on cancer metastasis, cell heterogeneity, and the development of personalized cancer immunotherapy. The F-SAW enabled ADE method (Figure 14) has several advantages, including convenience, rapidity, biocompatibility, and non-invasiveness, making it a powerful tool for single CTC encapsulation. Wei et al. [143] combined the ADE technique with a gelatin nanoparticle (GNP) modified substrate (GNP chip) that had multifunctional enzyme responsiveness to achieve on-demand release of single CTC. The ADE device was used to produce oxidized alginate-matrix metalloproteinase-9 (OA-MMP-9) droplets, which were controllable in size and accurately positioned on the GNP chip where the cell was adhered, allowing for enzyme-controlled biodegradation of the cell surface, followed by embedding of the single cell in alginate hydrogel through treatment with a calcium chloride (CaCl_2_) solution. The embedded cell maintained a good cell morphology in the hydrogel and could be efficiently recovered through a microcapillary. Li et al. [144] used the ADE technique to recover single CTC from the peripheral blood of cancer patients. The ADE was pulse-width-sensitive, with droplet diameters ranging from 350 to 550 μm, and adjusting the cell concentration and the pulse width duration enabled the maximum probability of single-cell encapsulation (11.61%). The encapsulated cells were subsequently labeled with three-color fluorescence and extracted by microcapillary for single-cell analysis. Li et al. [145] constructed a single CTC capture and encapsulation platform based on ZnO nanofibers and surface acoustic waves. Dense ZnO nanofibers provided additional binding sites, increasing the capture efficiency to 93.3%. The substrate was then dissolved with a low concentration of (25 mM) phosphoric acid to release 100% of the cells into an alginate solution, where the released cells remained active and proliferative. Finally, single CTC were encapsulated using the acoustic droplet ejection device, and the encapsulation rate of a single cell could reach 13% with a pulse width of 520 μs.

### 4.2. Construction of Tumor Microenvironment

**Figure 15:**
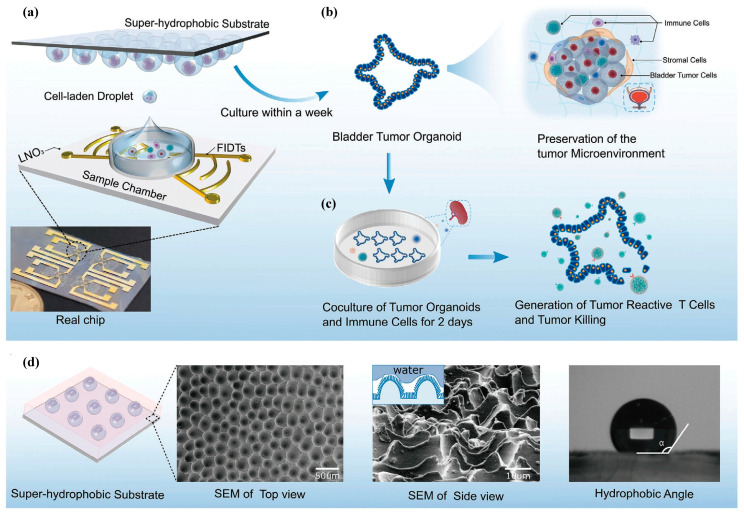
Working principle of ADE-based organoid printing platform. (**a**) Schematic illustrations of bladder tumor organoid formation, not drawn to scale. (**b**) Schematic representation of the organoid depicting the preserved immune microenvironment of the bladder tumor. (**c**) Schematic representation of bladder tumor organoid co-cultured with autologous immune cells derived from the spleen to generate tumor-reactive T cells. (**d**) Scanning electron microscopy (SEM) image and contact angle of the hydrophobic substrate. Reprinted from Gong et al. [147].

The current limitations of 3D bioprinting technologies is hard to precisely put bioinks in high-fidelity 3D structures and to print a sufficient concentration of multiple cell types with superior survival capabilities. This is due to the use of nozzles in existing bioprinters, which limits the printing resolution and leads to serious clogging issues when the nozzle size is below 100 µm as well as increased shear stress during the printing process. Acoustic droplet ejection (ADE) is a novel nozzle-free printing technology that reduces the maximum shear stress during the printing process by 2.7 times compared to 150 µm valve-based nozzles [79]. This enables the stable printing of high concentrations of cells or cell spheroids, with a cell survival rate (>94%) higher than those achieved using inkjet-based (>85%) or extrusion-based (40–80%) bioprinting methods. ADE allows for the printing of different types of bioinks and overcomes the limited printability of low-concentration gelatin methacryloyl (5% (*w*/*v*) GelMA) materials. By determining the position, flow characteristics (e.g., diffusion), and 3D shape of GelMA droplets, high-fidelity 3D constructs can be produced. Chen et al. [146] constructed a tumor microenvironment using acoustic droplet printing, which included a tumor spheroid and a high concentration of cancer-associated fibroblasts (CAFs). The dynamic tumor invasion function can be regulated by the interaction between tumor cells and CAFs. Gong et al. [147] combined acoustic droplet printing with hydrophobic substrates to produce a large amount of homogenous and highly viable bladder tumor organoids in vitro within a week (Figure 15). The generated tumor organoids contained all components of bladder tumors, including a variety of immune elements and tumor cells. Tumor-reactive T cells were induced in vitro by co-culturing the tumor organoids with autologous immune cells for two days. Chen et al. [150] used acoustic droplet printing to accurately encapsulate CAFs from colorectal cancer (CRC) patients in GelMA droplets and print them into 3D CAF microtissues. When the tumor organoids from the same patient were deposited, the migration and invasion of cancer cells from the tumor organoids to the 3D CAF microtissues were simulated. This was further studied using time-lapse imaging to investigate the dynamics of cancer invasion and its therapeutic response. Chen et al. [151] also used acoustic bioprinting to precisely arrange patient-derived microtissues (PDMs) composed of colorectal tumor and healthy organoid samples to reconstruct the original tissue structure for the understanding of histological, genomic, and phenotypic features of the primary tumor. By establishing the correlation between the invasion speed of organoid samples and the standardized diffusion speed of matched patients, this technique can help clinicians make better decisions regarding the treatment of CRC patients.

### 4.3. Drug Discovery and Cell Cryopreservation

**Figure 16:**
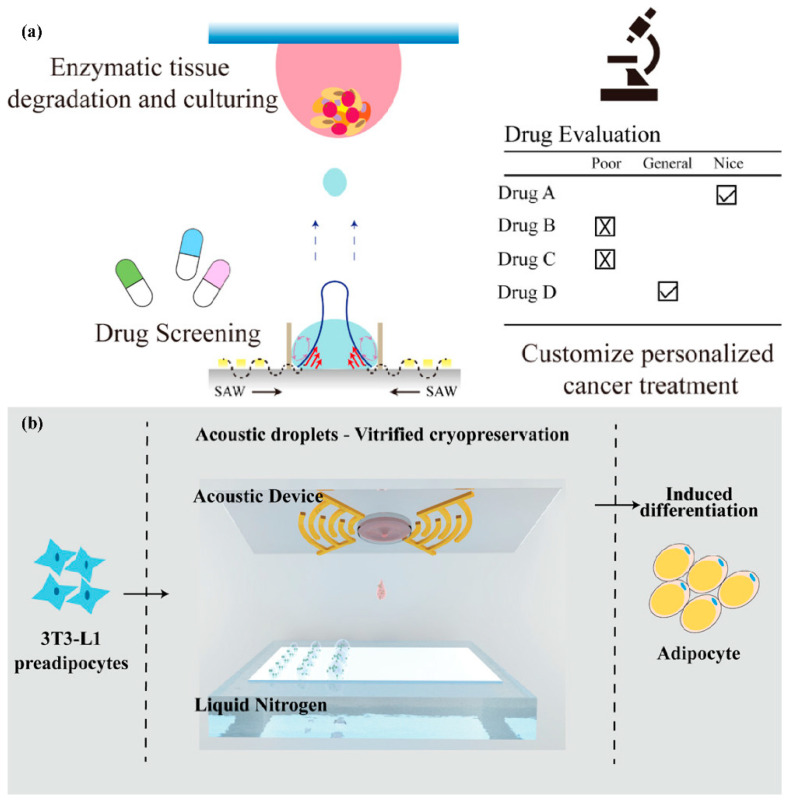
(**a**) Schematic illustration of ADE method for drug screening (not to scale). Reprinted from Xia et al. [148] (**b**) Schematic illustration of acoustic droplet vitrification method for cell cryopreservation (not to scale). Reprinted from Xia et al. [149].

High-throughput screening of cells is a critical step in nowadays’ disease research, precision medicine and drug development. However, traditional 2D cell models exhibit significant differences from the tumor microenvironment and lack low-consumption screening platforms for low cell numbers. Xia et al. [148] have established a rapid cell culture and drug screening platform using a superhydrophilic–superhydrophobic microarray platform combined with ADE technology (Figure 16a). By using hydrophilic and hydrophobic microarrays, cell suspensions can be automatically distributed into uniform droplets and construct tight 3D cell spheroids within 36 h at the same time. Subsequently, ADE technology precisely injects drug solutions of ∼pL to ∼nL volumes into the droplets, with the entire process finished in 20 milliseconds. By culturing and screening colon cancer cells from five patients, the best treatment method determined was highly similar to the actual treatment outcomes of the patients.

Low-temperature preservation of cells is a crucial step in current fields, such as translational medicine, regenerative medicine, reproductive medicine, and cell therapy. However, for certain rare cells with low numbers and cells lacking efficient and biocompatible preservation strategies, there are still significant challenges in practical applications. To address this issue, Xia et al. [149] utilized the acoustic droplet ejection (ADE) technology to encapsulate rare cells into droplets with volumes ranging from ∼pL to ∼nL, which were then deposited onto an array of droplets on a substrate (Figure 16b). By combining a cooling system within the droplet array substrate, rapid vitrification of hundreds to thousands of rare cells was achieved within a short period of time (approximately 2 s) on the basis of the high surface area-to-volume ratio of the droplets. This resulted in excellent cell viability (e.g., >85% for days, >70% for months), proliferation, and adhesion even after a prolonged period of time.

### 4.4. Chemical Synthesis and Protein Crystallography

**Figure 17:**
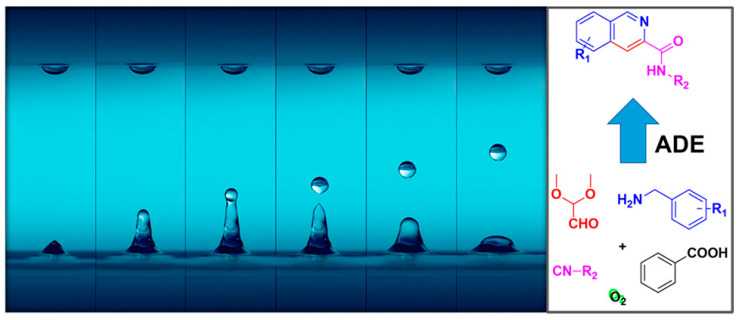
The application of ADE technology in chemical synthesis. Reprinted form Wang et al. [156].

ADE technology can be used for the automation, miniaturization, and acceleration of chemical synthesis, reducing resource consumption and waste while providing products in shorter times (Figure 17). This technology can transport nanoliter-scale droplets of reagents with precision and accuracy without contact-based dispensing techniques, avoiding potential sources of impurities. Due to the highly automated nature of the ADE platform, chemists are exposed to fewer hazardous chemicals, solvent vapors, and reaction risks, which is crucial for safety. Shaaban et al. [155] utilized ADE technology and rapid quality control techniques to perform high-throughput screening of chemical reactions at the nanoscale for the first time, developing an efficient synthetic reaction using an interrupted Fischer indole reaction combined with Ugi-type reactions, resulting in a bunch of attractive drug-like scaffolds. Wang et al. [156] designed and discovered a new synthesis method for isoquinoline using an nL droplet transfer component which utilized ADE technology, placing each compound in a discrete hole to automatically synthesize 384 random derivatives and monitoring their quality through SFC-MS and TLC-UVM analysis. The ADE method can quickly explore reaction systems to identify novel intermediates and drug scaffolds and can be scaled from nanometers to milligrams. Additionally, this method can be extended to include traditional and novel organic synthesis reactions, including transition metal-catalyzed reactions. Sangouard et al. [157] utilized ADE technology to prepare a 2700-membered targeted macrocycle library and tested it in the same microplate, saving additional transfer and separation steps, ultimately screening the library against the MDM2–p53 protein–protein interaction and generating micromolar and sub-micromolar inhibitors, providing insights for the development of a general strategy for macrocycle ligands.

ADE is also a promising approach for the manipulation of crystal formation via the precise addition of small volumes of crystallization reagents, chemical additives, or ligands. ADE optimizes crystal morphology and diffraction quality by facilitating acoustic transfer. This technique enables rapid vapor diffusion equilibration kinetics and efficient reagent usage, allowing for nanoliter-scale crystallization experiments. Consequently, this methodology holds great potential in promoting empirical discovery of crystallization conditions for proteins that rely on structure determination [158].

### 4.5. Acoustic Ejection Mass Spectrometry 

**Figure 18:**
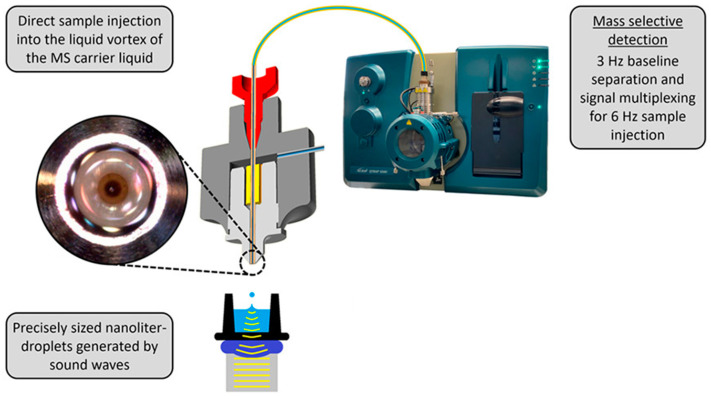
AEMS setup for contactless electrospray ionization mass spectrometry (ESI-MS)-based sample injection. Reprinted form Häbe et al. [160].

Acoustic ejection mass spectrometry (AEMS) technology uses sound waves to non-destructively separate and transport low-nanoliter volume sample droplets from a microplate to a continuous fluid transfer open port interface (OPI) for atmospheric pressure ionization (Figure 18). Combining an ultra-high throughput (UT) reader platform with acoustic droplet ejection-open port interface-mass spectrometry (ADE-OPI-MS) provides the necessary sensitivity and speed for high-throughput experiments (HTE). Dirico et al. [159] reported the application of ADE-OPI-MS in the fields of parallel medicinal chemistry and reaction screening, which offers very fast and thorough MS readings, minimal sample consumption, and quantitative detection of a broad range of chemical entities that can be directly coupled with high-density microdroplet plates. Equipped with high-resolution MS (HRMS), the instrument enables unambiguous identification of both small and larger (on-DNA compound) substrates. Häbe et al. [160] combined OPI with an improved acoustic droplet ejection (ADE) system by adopting AEMS technology, which greatly shortens the transfer distance between the OPI and ESI electrodes, and optimizes the throughput of the ESI-MS system. The pressure tests on ion suppression and system tolerance were carried out for unlabeled applications in simulating drug metabolism and pharmacokinetics (DMPK) analysis and high-throughput screening (HTS) activities. The results showed that this technology can achieve a maximum sampling rate of up to 6 Hz with minimal sample preparation and exhibits good signal stability and reproducibility. Liu et al. [161] described the sound wave droplet distribution component of the AEMS system, which includes sampling speed, droplet volume calibration, precision, repeatability, and different operating modes on the microplate. They proposed a “continuous infusion” mode to provide a low sample consumption steady-state signal and a concept of “barcode” mapping the microplate position to a specific mass spectrum signal by combining droplet modes.

## 5. Conclusions and Future Outlook

Acoustic drop ejection (ADE) technology, which utilizes sound waves to eject droplets from liquids, has become a widely used technique in various fields including biomedicine, materials science, chemical analysis, and high-precision coating. ADE allows for the accurate positioning of tiny droplets without touching the sample and offers advantages such as high throughput, high precision, non-contact, and integration with automated systems while saving samples and reagents. The technology relies on the production of local high-intensity sound pressure by a focused transducer to achieve rapid and stable ejection of droplets at volumes ranging from nanoliters to picoliters. However, traditional focused transducers, such as concave transducers, self-focusing acoustic transducers, and acoustic transducers with lenses, have limitations in terms of size, complexity, processing requirements, and difficulty in further increasing frequency, making them unsuitable for downsizing ADE systems. On the other hand, ADE technology based on surface acoustic waves (SAW) induction is simple in structure, biocompatible, and easy to implement at ultra-high frequencies using MEMS processes. It has therefore become a frontier technology in ADE. This review summarizes the basic theory, influencing factors, and application scenarios of SAW-enabled ADE technology with the aim of comprehensively understanding the technology and promoting its further development.

The review first introduces the basic theory and simulation verification of SAW-enabled ADE from three aspects: acoustic streaming theory, jetting induced by SAW, and simulation verification. After SAW contacts the liquid on the substrate, it enters the liquid in the form of LSAW, inducing SAW streaming effects. The streaming force is obtained by solving the SAW streaming equation using perturbation theory and retaining the second-order term and averaging over time. SAW-induced ejection requires that the droplet size be larger than the LSAW wavelength, the streaming Reynolds number Res be greater than 1000, and the jet Weber number ${\mathrm{We}}_{j}$ be greater than 0.1, with multiple droplet ejection occurring when ${\mathrm{We}}_{j}$ exceeds 0.4. The ejection process and changes in the internal and external fields can be visualized and simulated using LBM and CLSVOF, with the latter combining the advantages of LS method for accurately calculating curvature and capturing sharp interfaces and the VOF method for maintaining mass conservation.

The production of surface acoustic wave (SAW) jets is influenced by four main factors: fluid properties, device configuration, presence of channels or chambers, and driving signals. To achieve SAW droplet jetting, the size of the mother droplet must be greater than the wavelength of the SAW. In addition, the size of the mother droplet affects the size of the jet, which can be controlled by adjusting the power and modulation pulse time. When the viscosity of the liquid increases, the driving of SAW becomes more challenging due to the rise in acoustic attenuation, leading to a decrease in radiation pressure and an increase in streaming. The surface tension of the liquid affects the jetting height and volume, with greater surface tension leading to lower jetting height and larger volume. The device configuration includes material type, IDT type, and SAW excitation type. SAWs are mainly generated on piezoelectric single crystals and piezoelectric thin films, with LiNbO_3_ single crystals receiving widespread attention due to their high electromechanical coupling coefficient and low propagation loss. The 128°Y-X LiNbO_3_ produces Rayleigh SAWs that exhibit high coupling with fluids, making them widely used in microfluidic manipulation. Piezoelectric thin films, with their unique stretchability and integration with different substrates, expand the range of surface wave generation and application but require high power and exhibit significant thermal effects in fluid manipulation. Combining SAW with phononic crystals and other metamaterials provides additional ways to control fluid flow. Increasing the contact angle by treating the substrate with hydrophobic materials is a simple method to enhance driving ability. To achieve vertical jetting, symmetric IDTs are required to generate standing SAWs, and commonly used structures include C-ITDs, F-ITDs, and double straight IDTs. Although double straight IDTs have a simpler structure and save space, they exhibit less focusing performance than C-ITDs and F-ITDs due to aperture effects. Currently, C-ITDs and F-ITDs remain mainstream. In addition to Rayleigh SAWs, Lamb waves, SRBW, high-order waves, and SH-SAWs can be generated and applied to microfluidic manipulation using different substrate materials and sizes. High-order waves and SH-SAWs can excite liquid vertical jetting with only single IDT, but their coupling performance is still best with Rayleigh SAWs.

Based on the presence or absence of channels or chambers on the substrate, ejection can be divided into two types: sessile droplet ejection and ejection of liquid inside the channels. Sessile droplet ejection can overcome gravity and form a liquid bridge in an upward direction, while pendant droplets are affected by the same direction of gravity and radiation pressure. In the process of generating dispersed phase droplets in a continuous phase, liquid droplets inside a pipe are subjected to acoustic radiation force (ARF) at the interface between two fluids with different acoustic impedances, which creates a density gradient and induces deformation of the liquid–liquid interface. The size of the droplets can be adjusted by controlling SAW power, continuous phase flow rate, pulse duration, and channel geometry. Chambers are often used as liquid reservoirs placed next to the chip or on top of a focused acoustic transducer, which can be used to eject droplets into the air or any desired location. The direction of ejection can be adjusted through input signals of F-IDTs, but the ejection effect is affected by the stability of the liquid surface, chamber size, and liquid level. The application of acoustic valves provides the possibility of controlled release of droplets. The ejection of droplets is also affected by the power, frequency, and pulse duration of the input signal. Typically, as the input power increases, SAW streaming, vibration, transportation, jetting, or atomization of the liquid on the substrate occurs in sequence. The differences between jetting and atomization mainly depend on the relationship between the characteristic size of the droplets on the substrate and the wavelength of LSAW, which can also be explained from the perspective of the hydrophilic-hydrophobic angle of the substrate. A higher input frequency leads to a higher energy dissipation rate, a smaller amplitude of substrate vibration, a shorter LSAW attenuation length, and less energy reaching the free surface. Therefore, to achieve a driving effect at a lower frequency, the input power must be increased. By modulating the input signal, the driving effect of the droplets can be enhanced, the required driving power can be reduced, and the thermal effect of the substrate can be weakened. Moreover, different pulse durations can also be used to adjust the ejection size of the droplets. SAW-enabled ADE is widely applied in various fields such as bioprinting, chemical synthesis, drug analysis, and protein crystallography. This review focuses on its specific applications in the encapsulation of circulating tumor cells, construction of tumor microenvironments, drug discovery and cell cryopreservation, chemical synthesis and protein crystallography, and acoustic ejection mass spectrometry.

Although the SAW-enabled ADE technology has broad application prospects, there are still many issues worth studying. The existing theory still uses simplified analysis and does not describe or ignore many details. The reaction between acoustic waves and liquid is nonlinear, and the response involves time and length scales that differ by several orders of magnitude. It is more difficult to analyze and simulate the jet behavior in depth. Existing simulation technology ignores the heat transfer and evaporation of droplets, especially the thermal effect of SAW is more obvious on piezoelectric films, and the smaller the droplet, the more obvious the evaporation effect. Therefore, this simple neglect is obviously inappropriate. When interacting with higher viscosity liquids, most of the energy will be dissipated in the liquid, which is unfavorable for liquid jetting or even atomization. However, current research is still limited to a few low-viscosity liquids, and research on jetting problems of high-viscosity liquids is insufficient. Therefore, there is still a certain distance for the application of high-viscosity biological ink in bioprinting. At the same time, attention should also be paid to the influence of viscosity on thermal effects because cells are relatively sensitive to temperature and excessive temperature will reduce cell survival rate. Admittedly, hydrophobic treatment of substrates can significantly enhance jetting performance, but it is not clear how changing hydrophobicity (or contact angle) specifically affects the transmission and distribution of surface wave energy to enhance jetting performance. The thickness and material properties of hydrophobic films will also have an impact on devices (frequency, thermal effects, substrate amplitude, SAW leakage and dissipation, etc.), and determining how to choose the optimal contact angle poses a challenge to quantitative research on surface treatment. C-ITDs and F-ITDs clustered IDTs do not need to consider aperture size too much, but IDTs have a relatively large area. In some places where size is required, double straight IDTs may be more practical. However, there has been no suitable report on the reasonable design of its aperture. Preliminary experimental phenomena have found that aperture will affect the size of jetted droplets and substrate thermal effects, but this requires further experiments and theoretical support. Pure Lamb waves and pure R-SAW can be used as driving waveforms for droplet jetting. SRBWs that combine Lamb waves and R-SAW have also made significant progress in improving atomization effects. However, whether HYDRA can improve droplet jetting is also a question worthy of study; Sezawa wave has a higher sound speed and larger signal amplitude than R-SAW, but such waves are rarely used for droplet jetting. This also raises questions about whether other waves or various types of mixed waves can be applied to jetting and whether their performance can be improved or whether they can replace R-SAW in high-viscosity liquid jetting. The ultimate goal of jetting is to achieve continuous and stable jetting. Therefore, it is necessary to cooperate with liquid pumping or storage devices to provide a continuous source of liquid at an appropriate flow rate. However, current research strategies, such as capillary pumping, have a lower jetting frequency (5–25 Hz) while placing circular storage tanks on C-ITDs will gradually decrease the liquid level with the injection of liquid, which will cause unevenness in droplet injection. At the same time, too much liquid requires corresponding power consumption and substrate thermal effects will become more obvious accordingly; too little liquid requires continuous replenishment of liquid into the tank. Therefore, seeking a way to quickly replenish liquid to improve jetting frequency and stability has become an urgent need.

In summary, SAW-enabled ADE has great potential and value in bioprinting, chemical synthesis, material analysis and other fields due to its unique advantages. However, it also faces many problems in structural characteristics, fluid characteristics, driving characteristics, and other aspects. The aim of this review is to offer a basis for professional people inside and outside this field who are interested in ADE by gathering relevant information for thinking so as to promote further development of this discipline.

## Data Availability

No new data were created or analyzed in this study. Data sharing is not applicable to this article.
